# Rotary Wind‐driven Triboelectric Nanogenerator for Self‐Powered Airflow Temperature Monitoring of Industrial Equipment

**DOI:** 10.1002/advs.202307382

**Published:** 2024-01-19

**Authors:** Yi Li, Haocheng Deng, Haoying Wu, Yi Luo, Yeqiang Deng, Hongye Yuan, Zhaolun Cui, Ju Tang, Jiaqing Xiong, Xiaoxing Zhang, Song Xiao

**Affiliations:** ^1^ State Key Laboratory of Power Grid Environmental Protection School of Electrical Engineering and Automation Wuhan University Wuhan Hubei 430072 China; ^2^ Beijing International S&T Cooperation Base for Plasma Science and Energy Conversion Institute of Electrical Engineering Chinese Academy of Sciences Beijing 100190 China; ^3^ State Key Laboratory for Mechanical Behavior of Materials Shaanxi International Research Center for Soft Matter School of Materials Science and Engineering Xi'an Jiaotong University Xi'an 710049 China; ^4^ School of Electrical Power South China University of Technology Guangdong 510640 China; ^5^ Innovation Center for Textile Science and Technology Donghua University Shanghai 201620 China; ^6^ Hubei Engineering Research Center for Safety Monitoring of New Energy and Power Grid Equipment Hubei University of Technology Wuhan Hubei 430068 China

**Keywords:** airflow temperature sensing, industrial IoTs, smart power grid, triboelectric nanogenerator, wind energy harvesting

## Abstract

Heat dissipation performance is crucial for the operational reliability of industrial equipment, which can be monitored by detecting the wind or airflow temperature of the radiator. The emergence of triboelectric nanogenerators (TENGs) provides new routes for wind energy harvesting and self‐powered sensing. Herein, a rotary wind‐driven triboelectric nanogenerator (RW‐TENG) with soft‐contact working mode is newly designed to achieve tunable contact areas by utilizing the reliable thermal response of NiTi shape memory alloy (SMA) to air/wind temperature. The RW‐TENG can generate different triboelectric outputs under air stimulation with different speeds or temperatures, which is demonstrated as a power source for online monitoring sensors, self‐powered wind speed sensing, and airflow temperature monitoring. Specifically, a self‐powered sensor of wind speed is demonstrated with a sensitivity of 0.526 µA m^−1^ s between 2.2 and 19.6 m s^−1^, and a self‐powered monitoring device of high airflow temperature, which show relatively short response time (109 s), strong anti‐interference ability and outstanding long‐term durability. This study introduces an innovative route for real‐time detection of airflow temperature in wind‐cooled industrial equipment, showing broad application prospects for information perception and intelligent sensing of the industrial IoTs.

## Graphical abstract

A rotary wind‐driven triboelectric nanogenerator (RW‐TENG) is designed with tunable rotator area by incorporating NiTi shape memory alloy (SMA). The soft‐contact strategy delivers multifunctional applications to RW‐TENG, including power source, self‐powered wind speed sensor, and airflow temperature monitor. Airflow temperature sensing of oil‐immersed transformer radiator is demonstrated, bringing a new route for intelligent operation of next‐generation smart industrial equipment.

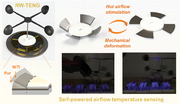

## 1 Introduction

Air or wind flow cooling strategy has been widely adopted for thermal management in various industrial equipment and personal devices, including electrical power transformers, generator sets, air conditioning, personal computers, etc.^[^ ^1^, ^2^, ^3^, ^4^, ^5^ ^]^ Real‐time monitoring of heat dissipation performance is essential for equipment operation and maintenance. For example, electrical equipment such as oil‐immersed transformers will experience oil temperature rise and aging due to a cooling system fault (e.g., the full shutdown of the cooler). Besides, the industrial Internet of Things (IoTs) has become a significant focus for the next‐generation smart industry, which relies on billions of distributed sensors to comprehend equipment information.^[^ ^6^, ^7^, ^8^, ^9^, ^10^, ^11^ ^]^ However, the power supply to the sensors is a choke point that limits the width and depth of state perception.

The invention and development of triboelectric nanogenerator (TENG) that convert varieties of low‐frequency, erratic, and ambient energy into electricity offer flourishing solutions for industrial IoTs.^[^ ^12^, ^13^, ^14^, ^15^, ^16^ ^]^ As one of the most important renewable energy sources, wind energy widely exists in aspects of industrial equipment.^[^ ^17^, ^18^, ^19^ ^]^ Numerous wind‐driven TENGs have been designed to power micro‐nano sensors, avoiding frequent battery replacement as well as complex wiring, especially in remote areas such as unmanned substations.^[^ ^20^, ^21^, ^22^, ^23^, ^24^, ^25^, ^26^, ^27^, ^28^, ^29^ ^]^ Moreover, regarding hot airflow energy harvesting, several methods for modifying triboelectric materials to break through wind‐driven TENG's working temperature limitations has been reported.^[^ ^30^ ^]^ For instance, *Tao* et al. proposed a fluorinated polyimide film with doped BaTiO_3_ nanoparticles demonstrating high surface charge density (200 µC m^−2^) and thermal charge stability.^[^ ^31^ ^]^ The feasibility of active‐sensing of physical parameters, including wind speed, and direction was also reported.^[^ ^17^, ^32^ ^]^ However, it remains challenging to realize self‐powered monitoring of the hot airflow temperature that can reflect industrial instruments' heat dissipation performance. Compared to the direct detection of heat dissipation medium (such as oil), airflow temperature detection belongs to a non‐contacting means that can avoid the insulation defects and maintenance challenges caused by built‐in sensors.^[^ ^33^ ^]^ Currently, air/wind temperature monitoring is commonly realized by commercial thermometers.^[^ ^34^ ^]^ Designing TENG with high response capability for self‐powered air/wind temperature detection is significant for realizing smart industrial IoTs.

Shape memory materials (SMMs), as reliable materials with thermal‐response performance, are capable of preserving a temporary shape (macroscopic and microscopic) and recovering to its original state during heat stimulation.^[^ ^35^, ^36^, ^37^ ^]^ SMMs can endow devices with shape‐reconfigurability and performance stability, which act as active layers for TENGs have been demonstrated for self‐powered sensors or alarms.^[^ ^37^, ^38^, ^39^, ^40^ ^]^ For example, *Xiong* et al. achieved the active sensing of water temperature using the electrospun micro‐structured shape memory polymer (SMPs) based TENG with thermal‐induced recovery of hydrophobicity and output voltage dependent on the surface roughness.^[^ ^39^ ^]^ *Yang* et al. reported a thermal‐driven TENG as the smart switch for turning on/off the heating via human movement.^[^ ^40^ ^]^ Compared to SMPs, shape memory alloys (SMAs) deliver higher mechanical strength and shaping ability, offering more options for industrial IoTs that have higher requirements for the stability and reliability of sensing devices.^[^ ^41^ ^]^ For air/wind temperature detection by TENG‐based self‐powered sensors, the low start‐up torque, high charge density, and high durability are crucial for device sensitivity and environment adaptivity.^[^ ^42^, ^43^, ^44^, ^45^ ^]^ Integrating SMAs with high thermal response and deformation reliability to design a rotating mode TENG triggered by wind, with tunable electrical outputs under different temperatures of air/wind, is a promising solution to achieve self‐powered wind temperature sensing.

Herein, a rotary wind‐driven TENG (RW‐TENG) with soft‐contact mode was designed using NiTi alloy with thermally adjustable contact area with the coupled triboelectric material. Upon thermal air/wind stimulation, the top active layer of fur supported by NiTi alloy can change its contact area with the bottom layer, demonstrating a “curved” state at normal temperature wind and “flattened” state under hot wind (>40 °C). As a power source at room temperature, the RW‐TENG delivered an average power density of 140 mW m^−2^ at the wind speed 12 m s^−1^, which can serve as a self‐powered sensor of wind speed with the sensitivity of 0.526 µA m^−1^ s between 2.2 and 19.6 m s^−1^. Besides, the RW‐TENG exhibited superiority in detecting the high airflow temperature, which show relatively short response time (109 s), strong anti‐interference ability and outstanding long‐term durability. As a proof of concept, an RW‐TENG‐based self‐powered sensing system was established to provide wireless monitoring and alarming of airflow temperature in an oil‐immersed transformer. This work validated a new approach for real‐time detection of airflow temperature, showing broad application prospects for information perception and intelligent sensing of the industrial IoTs.

## 2 Results and Discussion

### 2.1 Shape Memory Alloy‐Based RW‐TENG with Tunable Rotator Area

**Figure 1:**
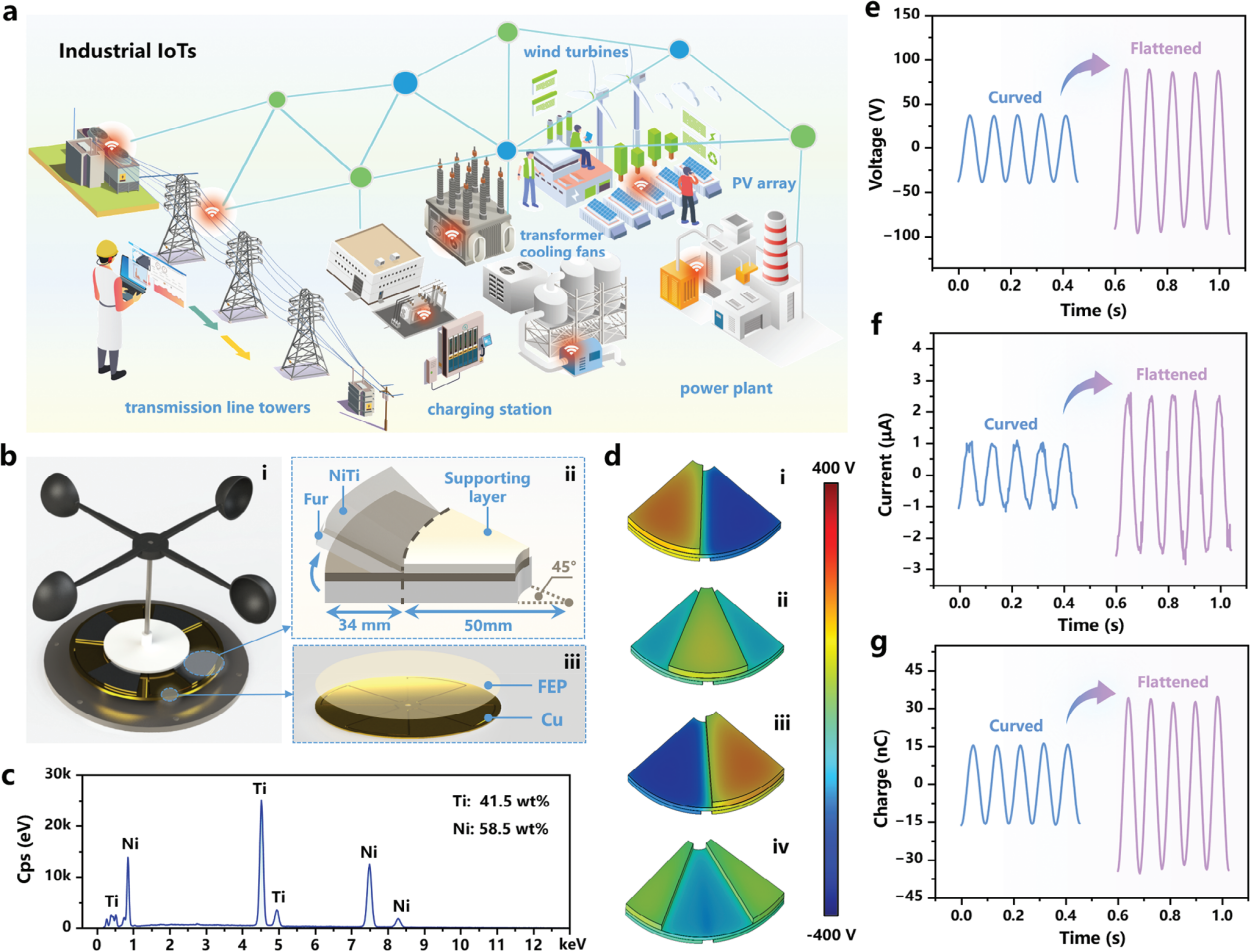
Shape memory alloy‐based RW‐TENG with tunable rotator area. a) Schematic illustration of a smart industrial IoTs system with wind‐driven TENGs platforms for state perception and real‐time monitoring of the electrical equipment. b) Structural schematic of the RW‐TENG. The enlarged view of the rotator demonstrates the tunable triboelectric contact area enabled by NiTi alloy with temperature response capability. c) EDS spectrum of the shape memory NiTi alloy. d) Potential distributions for the RW‐TENG obtained by electrostatic field simulation. Typical waveforms of the triboelectric e) open‐circuit voltage, f) short‐circuit current, and g) short‐circuit transferred charge with curved SMA and flattened SMA.

**Figure** **1**a briefly depicts the emerging strategy for state perception and real‐time monitoring of electrical equipment to realize the industrial IoTs. Wind‐driven TENGs could be widely deployed on transmission line towers, wind turbines, transformer cooling fans to harvest wind energy as well as realize self‐powered detection of the wind speed, direction, and temperature, safeguarding the operation of the power equipment.^[^ ^46^, ^47^, ^48^, ^49^, ^50^, ^51^ ^]^ The wind temperature detection strategy based on TENG belongs to a non‐contact detection mode, which requires high response and stability.

Herein, a novel RW‐TENG with a tunable rotator area enabled by NiTi alloy was proposed in Figure 1b and Figure S1 (Supporting Information). The stator, rotator, and wind cup framework were prepared through 3D printing. The patterned Cu electrodes were covered with 320 mesh sandpaper‐polished fluorinated ethylene propylene (FEP) film to form the stator (Figure 1b(iii)). Sandpaper grinding that created micro‐nano grooves on the FEP surface offered superior triboelectric performance by expanding the effective contact area, according to the SEM morphology and AFM surface roughness images given in Figure S1 (Supporting Information). The rotator triboelectric layer adopted a soft‐contact medium of trimmed rabbit fur (thickness of 20 mm) with rich fluffs, providing a large area to ensure full contact with the FEP film. It mainly consists of protein that has abundant oxygen‐containing groups, offering a significant number of electrons to FEP film during operation.^[^ ^25^, ^52^ ^]^ Besides, the soft‐contact mode can effectively reduce the start‐up torque as well as decrease the device's abrasion. Four sectors of rabbit fur, each angled at 45 °, were pasted beneath the same sized NiTi SMA. When exposed to high temperatures at the phase transition temperature (*T*_g_), the NiTi SMA briefly transitions from the martensitic phase to the austenite phase, and the *T*_g_ primarily depends on the composition ratio of Ni and Ti.^[^ ^53^ ^]^ As shown in Figure 1c and Figure S2 (Supporting Information), the SMA demonstrates uniform element distribution, where Ni accounts for 41.5 wt.% and Ti accounts for 58.5 wt.%. Moreover, differential scanning calorimetry (DSC) curve reveals that the *T*_g_ of NiTi SMA is ≈40 °C. The martensitic‐austenite transformation happens above the austenite start temperature (*A*_s_, 37 °C), and all deformation completes below the austenite finish temperature (*A*_f_, 47 °C). The disc‐shaped supporting layer was made of light 3D‐printed polylactic acid (PLA) with a radius of 50 mm, ensuring the rabbit fur sectors with a larger radius of 84 mm can be freely controlled by the NiTi SMA layer in shape.

Notably, the rotator triboelectric layer was designed to be tunable in contact area with the stator layer, driven by the thermally deformable NiTi alloy. Precisely, the shape memory NiTi alloy can be twisted and shaped together with the rabbit fur at room temperature, as shown in Figure 1b(ii), which can quickly regain its set shape (the flattened state) as the temperature reaches the *T*_g_ (the predetermined warning temperature of 40 °C), enabling enlarged contact area of the rabbit fur with the stator. A larger effective contact area contributes more tribo‐charges, bringing higher output performance. Therefore, a correlation between airflow temperature and triboelectric output can be built to realize self‐powered temperature sensing.

The working mechanism of RW‐TENG is illustrated as follows. The oncoming airflow passes through the hemispherical wind cups and alternately pushes them to revolve around the aluminum shaft. The rotator will rotate in the same direction, leading to triboelectric effect between the FEP film and rabbit fur. As a result, the rabbit fur is positively charged and the FEP film obtains electrons, and the tribo‐charges are alternately induced on the Cu electrodes. The electric potential distribution of RW‐TENG in a typical tribo‐cycle was investigated under the open‐circuit condition through electrostatic field simulation, and the triboelectric performance was provided, including open‐circuit voltage (*V*_oc_), short‐circuit current (*I*_sc_), and short‐circuit transferred charge (*Q*_sc_) with curved SMA and flattened SMA under the same rotation speed of RW‐TENG. As demonstrated in Figure 1e–g, it is evident that the output voltage and charge waveforms are both flawless sine waves, while the current waveform has tiny discharge pulses based on the sine wave. RW‐TENG with flattened SMA showed an output voltage of 87.5 V, short‐circuit current of 2.57 µA, and transferred charge of 33.51 nC, which is 2.36 times, 2.52 times and 2.14 times higher than those of curved SMA (37.0 V, 1.02 µA, 15.66 nC). The similar working waveforms with SMA under different states confirm the same working mechanism of RW‐TENG with conventional freestanding working mode of wind‐driven TENGs,^[^ ^54^ ^]^ as the potential distributions shown in Figure 1d and the established model shown in Note S1 (Supporting Information).

### 2.2 Optimization and Electrical Performance of RW‐TENG

To better adapt to the outdoor ambient wind, outstanding working performance such as low start‐up torque, high charge density, high durability to work even under harsh conditions is required for RW‐TENG.^[^ ^21^, ^55^ ^]^ Besides, high sensitivity to the physical parameters (temperature, speed, etc.) is also pursued to form airflow sensing system without an external power supply. Herein, we designed the soft‐contact mode for RW‐TENG. The soft‐contact medium (rabbit fur) exhibits diverse macro, micro surface morphology under various conditions, which may be the primary factor influencing the output. The RW‐TENG with flattened SMA possessing diverse structure parameters was fabricated to clarify the impact further.

**Figure 2:**
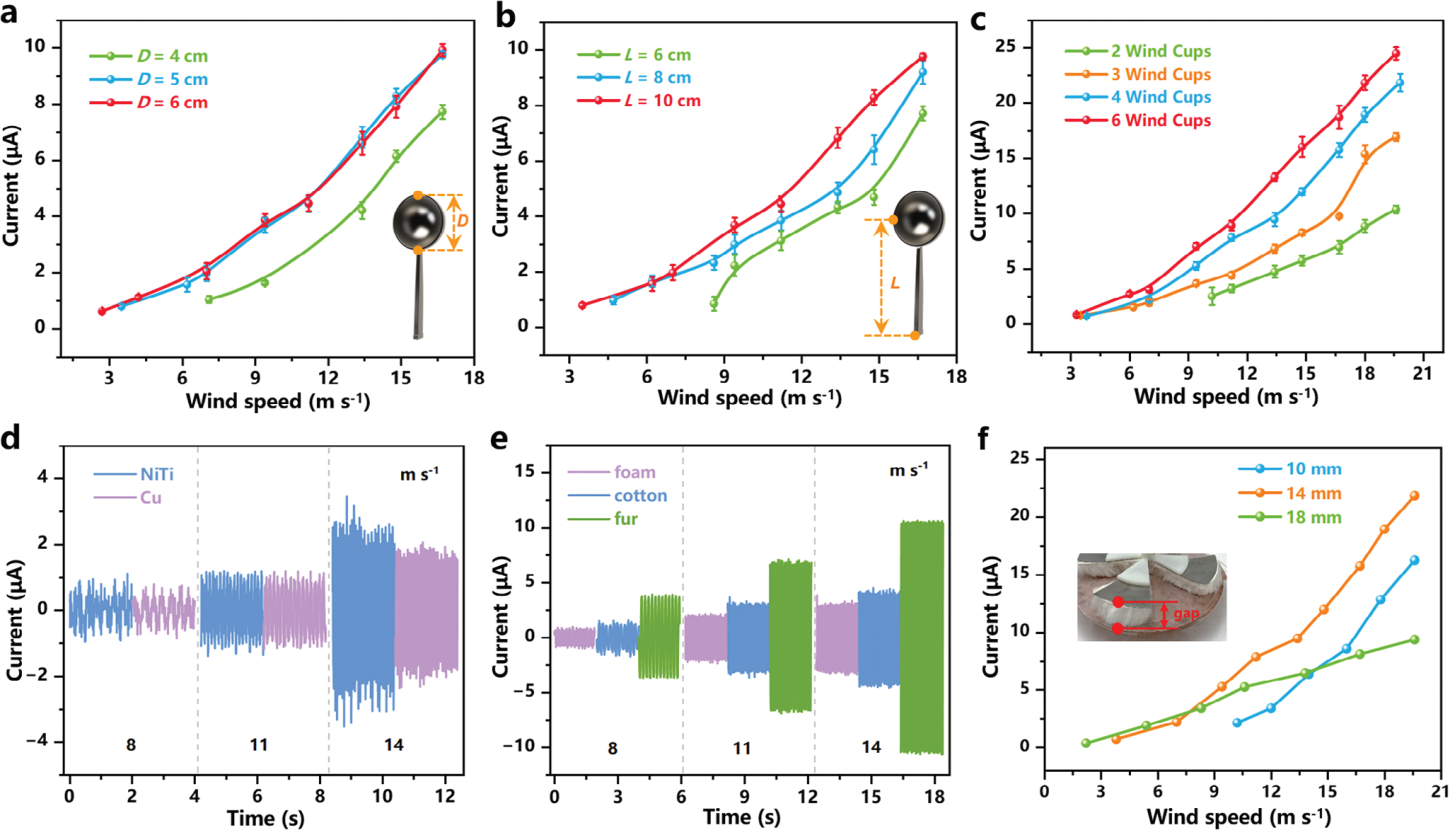
Structural optimizations of the RW‐TENG. The short‐circuit current of the RW‐TENG (air gap: 14 mm) with wind cups of different a) diameter, b) arm length, and c) number. Especially, not all the windward areas are blown by wind when *D* is 6 cm in this work (diameter of blower's air outlet: 5.5 cm). The short‐circuit current of the RW‐TENG in d) direct‐contact mode and e) soft‐contact mode. f) The short‐circuit current of the RW‐TENG with different air gaps.

Functioning as the wind energy collection module, the aerodynamic performance of the wind cups displays a crucial impact on the device.^[^ ^43^, ^56^ ^]^ Groups of lightweight wind cups (Figure S3, Supporting Information) were created through 3D printing to assess the impact of their parameters (diameter, arm length, and number) on the triboelectric output. The as‐fabricated RW‐TENG exhibited a start‐up wind speed of 7.1, 3.5, and 2.7 m s^−1^ with the wind cup diameter (*D*) of 4, 5, and 6 cm, indicating an inverse proportion (**Figure** **2**a).

The RW‐TENG also showed increasing current for the three sizes of wind cups as the wind speed increased. The variation of plotted current curves can be attributed to the different torque inputs from the wind cups. Equation 1 further illustrates the relationship between the input torque (*M*_1_), total windward area of wind cups (*S*_c_), wind speed (*v*_w_), and RW‐TENG rotation speed (*n*) (rpm).^[^ ^56^ ^]^

(1) $$M_{1}=\;k\times S_{c}{\left(v_{w}-\frac{\pi R}{30}n\right)}^{2}\times L$$

where *k* is the proportionality factor (associated with the air density), *L* is the arm length of wind cups (insert of Figure 2b). In addition, the resistance torque is unavoidable during the operation, which balances the input torque when the RW‐TENG rotates at a constant pace. The resistance torque (*M*_2_) is demonstrated by Equation 2:^[^ ^43^ ^]^

(2) $$M_{2}=M_{r}\;+M_{f}+M_{e}$$

where *M*_r_ is the rotational friction torque generated by the Al shaft, *M*_f_ is the torque generated by the friction between the dielectric, and *M*_e_ is the electrostatic torque generated by the mutual attraction of tribo‐charges. From Equation 1, larger *D*, which means larger *S*_c_, accordingly yields a larger *M*_1_. The RW‐TENG starts up after overcoming the resistance torque. Although factors including charge accumulation will increase *M*_2_ as the wind speed increases, *M*_1_ will increase faster, as will the output.

In addition, *I*_sc_ increased from 4.7 to 8.29 µA as the arm length of wind cups changed from 6 cm to 10 cm (wind speed at ≈14.8 m s^−1^), while the corresponding start‐up wind speed decreased from 8.6 to 3.5 m s^−1^ (Figure 2b). According to Equation 1, since *n* is low and the resistance torque to be overcome during starting is certain, the start‐up wind speed is inversely proportional to *L*. The output current demonstrated an upward trend with increased arm length and was likewise the case under high wind speeds. However, the result contrasts several reported works.^[^ ^43^, ^56^ ^]^ High‐density aluminum alloy was commonly employed to manufacture wind cups, resulting in excessive mass and resistance torque with the increase of *L*. Longer arm length also lowered the current by reducing the angular velocity of wind cups while maintaining the linear velocity. In this work, wind cups made of light PLA (the sparse filling technique used during 3D printing) offer a constant mass of the device, i.e., the constant resistance torque. Consequently, there was still an upward current trend as the *L* increased. As shown in Figure 2c, the start‐up wind speed was extremely high (10.2 m s^−1^) with two wind cups on the shaft sleeve and visibly dropped to 3.5 m s^−1^ subsequently as the number rose over three. No considerable decrease was observed with more wind cups. The output current increased from 10.4 to 24.48 µA (wind speed at ≈19.6 m s^−1^) with the number of wind cups increased from 2 to 6. However, too many wind cups will also reduce the output due to excessive mass. To summarize, 4 wind cups (*D* = 5 cm, *L* = 10 cm) were selected as wind energy harvesting modules because of excellent output efficiency and economy.

Various active triboelectric layers have been coupled with RW‐TENG to illustrate the advantages of the soft‐contact work mode. Figure 2d and Figure S4a (Supporting Information) depict the output of wind‐driven TENG with direct‐contact mode. When NiTi alloy was directly employed as the positive triboelectric layer (rabbit fur removed in Figure 1b(ii)), the output current demonstrated a slight improvement over that of Cu foil, while the transferred charge was approximately equal (≈20 nC). In addition, the rough particles on the stator surface interfered with the friction between the metal and the polymer, damaging the stator's triboelectric layer and leading to unstable operation and waveform distortion. The RW‐TENG demonstrated noticeably superior output when soft and porous materials (foam, cotton, and rabbit fur) were employed (Figure 2e; Figure S4b, Supporting Information). The highest output (current of ≈10.5 µA, charge of ≈120 nC) was achieved when FEP was coupled with the rabbit fur, verifying that the rabbit fur possesses the best tribo‐positive performance. Thereafter, we selected FEP (polished by 320 mesh sandpaper, 100 µm) and rabbit fur (trimmed, 20 mm) as the tribo‐pair for further performance evaluation of the soft‐contact RW‐TENG.

In addition, the air gap of the stator and rotator can be manually adjusted according to diverse application scenarios. Figure 2f demonstrates the short‐circuit current of RW‐TENG with air gaps of 10, 14, and 18 mm, dependent on the wind speed. A relatively constant airflow usually exists around the cooling system of large industrial equipment such as the power transformer. The 14 mm gap was the reasonable distance for RW‐TENG to exhibit the best performance in general circumstances with low start‐up wind speed (3.5 m s^−1^) and high output (≈22 µA under 19.6 m s^−1^). When the air gap was less than 14 mm, the increased pressure and frictional resistance between the triboelectric layer obstructed the start‐up of the device (start‐up wind speed of 10.2 m s^−1^ under 10 mm air gap), preventing the RW‐TENG from being applicated at low wind speed. The larger air gap (more than 14 mm) also brings output sacrifice due to insufficient contact. However, the short‐circuit current corresponding to the 18 mm air gap exhibited significantly more excellent linearity with variations in wind speed (*R* ^2^ = 0.995) than the 14 mm air gap (*R* ^2^ = 0.942). Coupled with the broader wind speed band (2.2–19.6 m s^−1^), the as‐fabricated RW‐TENG illustrated broad prospects in applying ambient wind speed sensor.

**Figure 3:**
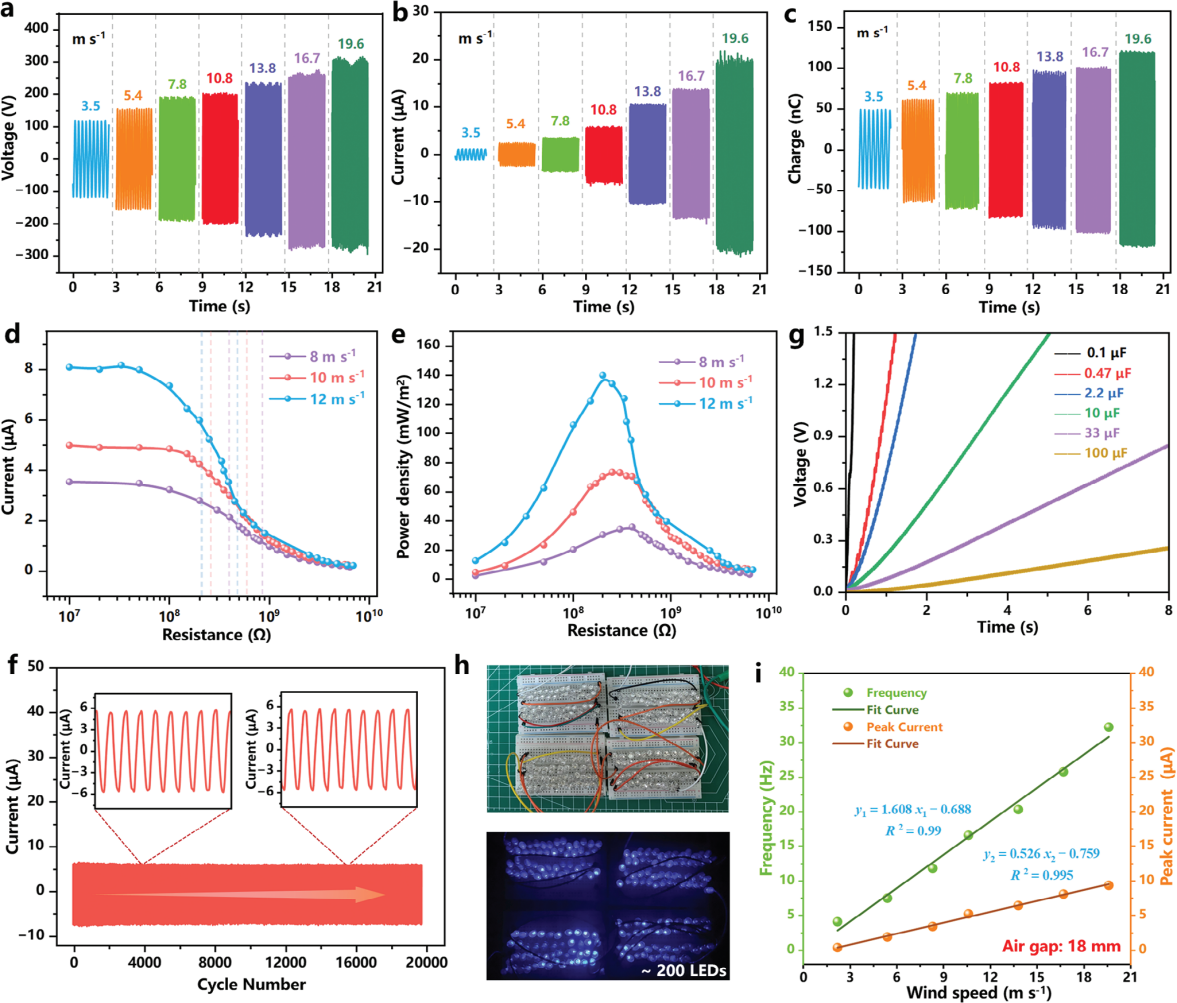
Triboelectric performance and application of RW‐TENG. The triboelectric a) open‐circuit voltage, b) short‐circuit current, and c) transferred charge of the optimized RW‐TENG under various wind speeds. Dependence of the d) short‐circuit current and e) peak power density of the RW‐TENG loaded with various external resistors. f) The output durability of the RW‐TENG under continuous measurement of ≈20 000 cycles. g) Charging curves of various capacitors by RW‐TENG at the wind speed of 10 m s^−1^. h) Demonstration of the RW‐TENG to power 200 LEDs. i) Measured relationship between the short‐circuit current, frequency and wind speed.

The electrical output including the open‐circuit voltage, short‐circuit current, and transferred charge quantity of the optimized RW‐TENG (air gap: 14 mm) were systematically measured (**Figure** **3**a–c) under the full wind speed band. The peak values of *V*_oc_, *I*_sc_, and *Q*_sc_ increased from 117.43 V, 1.13 µA, 49.11 nC to 315.43 V, 21.85 µA, 121.47 nC as the wind speed increased from 3.5 to 19.6 m s^−1^. The accelerated charge transfer between two electrodes of the RW‐TENG intrinsic capacitance was responsible for the rapid growth trend of *I*_sc_. Relatively smooth growth was observed in *V*_oc_ and *Q*_sc_. When RW‐TENG was just started, the straight needle hair end of the rabbit fur bent and fit along the FEP during the rotation, leading to the fluff wrapping. The bent straight needle hair also increased the rotator area equally, which enabled the rabbit to bridge between the electrodes. The gradually rising buoyant could elevate the straight needle hair end with increased wind speed. By virtue of the exposed fluffy structure, tribo‐charges started to build up and the likelihood of an electrode bridge diminished. Under various conditions, diverse macro and micro surface morphology endowed the as‐fabricated TENG with characteristics different from freestanding RW‐TENG in a direct‐contact mode, namely the upward trend of the *V*_oc_ and *Q*_sc_, rather than a saturated trend.

In addition, the load capacity performance of the optimized RW‐TENG (air gap: 14 mm) was investigated by connecting various load resistors (The calculation method of the output is shown in Note S2 (Supporting Information). The RW‐TENG showed a decreasing output current as the external load increased, which reached the peak power density of 140 mW m^−2^ at the wind speed of 12 m s^−1^ (Figure 3d,e). It also revealed a decreasing matching resistance from 400 MΩ (at 8 m s^−1^) to 200 MΩ (at 12 m s^−1^), in that the higher frequency reduced the RW‐TENG intrinsic impedance. The large intrinsic impedance similar to previous works^[^ ^25^, ^57^ ^]^ may be attributed to the increased average dielectric thickness due to the large air gap and the loose structure on the surface of rabbit fur, which was further clarified in Note S2 (Supporting Information). The linear area of the current curve (see the dotted line in Figure 3d) proved the feasibility of the RW‐TENG employed as a power source with adjustable internal impedance under different wind speeds. Such a RW‐TENG sustained ≈20 000 cycles and no apparent attenuation of the output current was observed (Figure 3f), confirming the outstanding structure reliability and output durability endowed by the soft‐contact strategy.

The RW‐TENG demonstrates promising application potential as a power supply or self‐powered wind speed sensor. The generated electricity can be stored by different electrolytic capacitors. As shown in Figure 3g, a capacitor of 0.1 µF can be charged to 1.5 V within only 0.2 s. The higher the wind speed is, the shorter the charging time is (Figure S5, Supporting Information). After rectification, 200 commercial LEDs can be obviously lit up under the wind speed of 10 m s^−1^ (Figure 3h). One of the fascinating merits of the RW‐TENG is that a robust linear relationship between electrical signals (short‐circuit current) and wind speed stimuli exists, as shown in Figure 3i and Figure S6 (Supporting Information). The *R* ^2^ values of the peak current and frequency curves reached 0.995 (with a sensitivity of 0.526 µA m^−1^ s) and 0.99 respectively under an air gap of 18 mm, which changed to 0.922 and 0.967 under 14 mm. Furthermore, the sensitivity of the self‐powered wind speed sensor with curved SMA was also investigated, as illustrated in Figures S7 and S8 (Supporting Information). RW‐TENG showed a lower start‐up wind speed (1.8 m s^−1^) because of the smaller stator‐rotator contact area and the 18 mm air gap in the contact part. The output current increased from the initial 0.74 µA to the final 3.79 µA as the wind speed increased to 19.6 m s^−1^, and the *R* ^2^ values of the peak current and frequency curves reached 0.990 and 0.966, respectively. The sensitive response at full wind speed band enables RW‐TENG with curved SMA as the self‐powered wind speed sensor as well, with a sensitivity of 0.15 µA m^−1^ s to identify faults in the cooling system instantly, alarming promptly when the cooler or fans stop blowing.

### 2.3 RW‐TENG for Self‐Powered Airflow Temperature Monitoring

**Figure 4:**
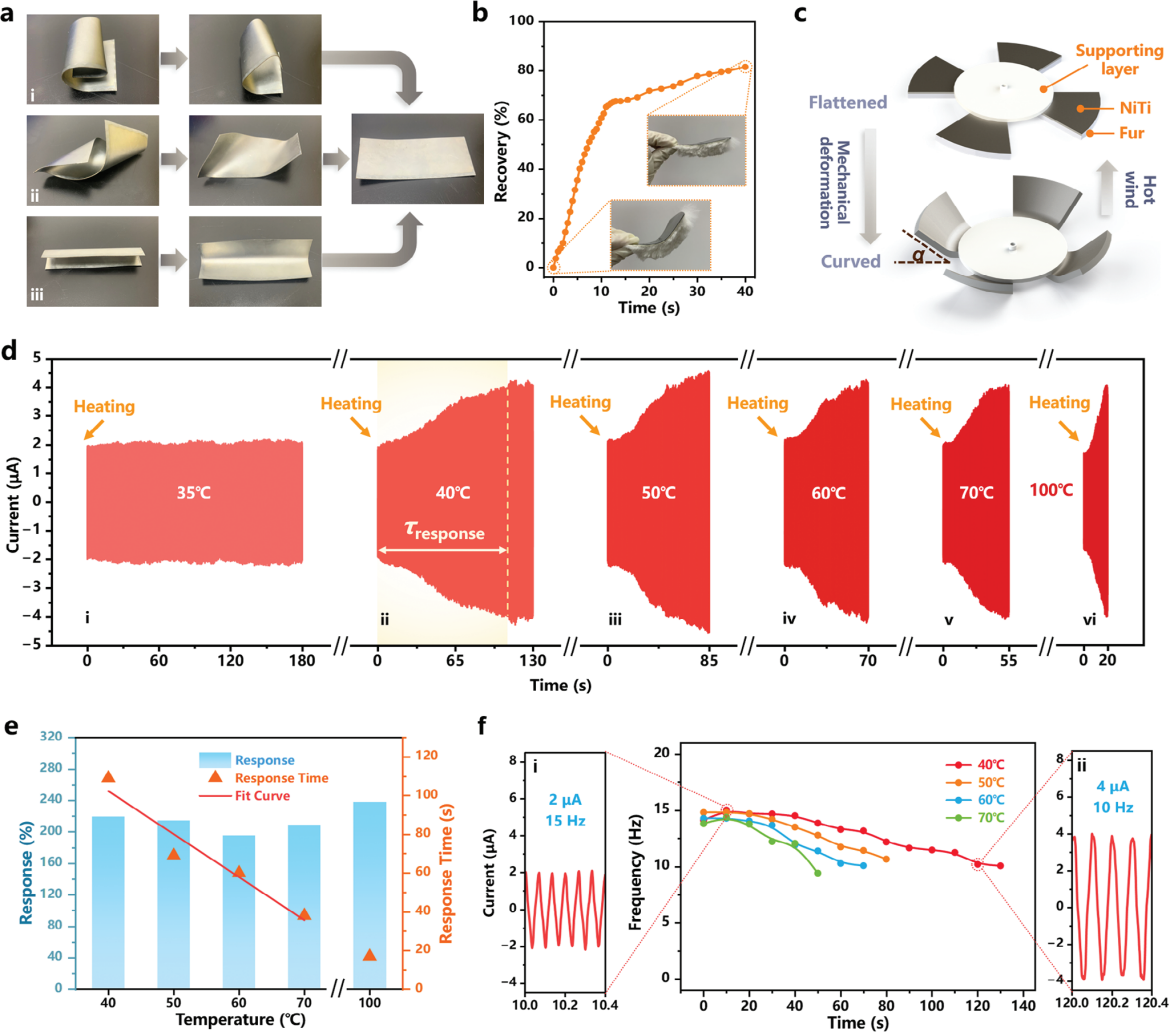
RW‐TENG for self‐powered airflow temperature sensing. a) The macroscopic shape memory effect of NiTi alloy. b) The recovery ratio of a rotator sector as a function of the heating time under 50 °C airflow. Insets are photographs of the rotator sector at the initial state and the recovered shape at 40s. c) Schematic transformation of the TENG between “curved” and “flattened” states. d) The current response of the RW‐TENG toward various wind temperature stimulations. e) Current response (*R*_C_) and response time (*τ*_response_) of RW‐TENG toward various wind temperature stimulations. f) Frequency response of the RW‐TENG toward various wind temperature stimulations.

A real‐time self‐powered high airflow temperature monitoring device is realized based on the RW‐TENG. **Figure** **4**a demonstrates the macro shape alteration of NiTi alloy. The sample (4 cm × 10 cm, Ni 58.5 wt.%, *T*_g_ ≈40 °C) was drastically twisted along the minor axis i), the diagonal direction ii), and the major axis iii) in turn, creating various shapes (i.e., the temporary shape) at room temperature (25 °C). After heating for 10 s under 80 °C airflow, it fundamentally returned to the original flattened state (i.e., the restored shape) regardless of its temporary shape. The NiTi alloy can also maintain its restored shape after cooling at room temperature, indicating excellent SME in all directions.

To further evaluate the macro deformation recovery performance, NiTi alloy was cut into a sector using a laser cutting machine with trimmed rabbit fur pasted below (according to the dimension displayed in Figure 1b(ii)). It was then twisted with an initial angle (*α*_0_) of 68.4 °, and the angle variations were continuously observed upon heating with 50 °C airflow for 40 s. As shown in Figure 4b, 70% recovery was observed within ≈18 s, and 81.6% recovery was observed when heating stopped (*α*(40 s) of 12.6 °), exhibiting a comparable shape‐restoring capability of the rotator material. Such four rotator sectors with peculiar thermal‐induced properties were fixed underneath the PLA supporting layer, creating the tunable rotator area. The rotator area that vertically aligned to the supporting layer was always in contact with the stator, while the exposed part was twisted at a certain angle (Figure 4c). The rotator demonstrated the minimum area (through prior mechanical deformation) when the RW‐TENG operated under normal temperature (“curved” state), and the maximum area was observed (after a period of recovery) under high‐temperature airflow (“flattened” state).

Triboelectric performance was explored for the thermal‐induced tunable area rotator (initially twisted angle *α*_0_ of 60 °). An 18 mm air gap was adopted for higher wind speed sensitivity, and 10 m s^−1^ wind was used to simulate the constant hot airflow. An instantaneous increase in heat source was realized by the hot wind gun (35–100 °C), aligned to the RW‐TENG rotator. Current response waveforms of the RW‐TENG exposed to various wind temperature stimulations (35, 40, 50, 60, 70, and 100 °C) are presented in Figure 4d (the entire current waveform with expanded data points is shown in Figure S9, Supporting Information). The output showed no response toward 35 °C airflow as the NiTi alloy remained in the martensite phase of orthogonal or monoclinic crystal at a temperature lower than *T*_g_. A remarkable response was observed toward 40 °C airflow (the ultimate transformer operating environment), and the entire waveform presented an “S” curve. Noticeably, there existed a transient decay in the output when the hot wind gun started initially due to the tribo‐electrons jumping into the air (the thermionic emission effect).^[^ ^31^, ^58^ ^]^ The output current then demonstrated an upward trend with the continuous heating, suggesting that the NiTi alloy realized an austenite phase transformation. Considering the rotator recovery trajectory (Figure 1b(ii)), the increase in triboelectric area corresponding to the per reduced twisted angle became greater (namely more tribo‐charges) when RW‐TENG approached the “flattened” state. Therefore, the current growth proceeded to pick up speed, which presented a lower half of “S”. However, the transformation rate into the austenite phase substantially decreased once the rotator recovery ratio reached ≈70% (Figure 4b). The output current curve demonstrated an upper half of “S” as a result (sluggish pattern of growth) until no significant distortion occurred. Physical images of the rotator state were captured to clarify the waveform further, as shown in Figure S10 (Supporting Information). During operation, the SMA along with the pasted rabbit fur gradually transitioned from the “curved” state to the “flattened” state under the hot wind stimulation above *T*_g_. Similar “S” curves were observed as well with the wind stimulations above 50 °C. In addition, temperature distribution images of RW‐TENG during long‐term operation of 35 min under the “curved” state were captured by an infrared thermal imaging camera, as shown in Figure S11 (Supporting Information). There was no obvious temperature change of the triboelectric layers after continuous operation for 35 min, and there was no noticeable temperature difference between the triboelectric layers and components such as the wind cups or Al shaft. It suggested that the long‐term friction would only produce a modest amount of heat and have no appreciable impact on the SMA's condition.

The RW‐TENG exhibited relatively rapid response time (*τ*_response_, the time spent on reaching 90% of the maximum current response in a single heating test), which demonstrated a sixfold decreasing trend (from 109 to 17 s) with the airflow temperature rising from 40 to 100 °C. It suggested that the increased airflow temperature could speed up the recovery process. The linear fitting between the response time and airflow temperature was further conducted (Figure 4e), delivering a sensitivity of 2.22 °C s^−1^ for the airflow temperature between 40 to 70 °C. Therefore, the response time (speed) as the indicator is capable of reflecting the wind temperature. Besides, RW‐TENG delivered a superior current response (*R*_C_, defined in Experimental Section) of 219.16% under 40 °C airflow, indicating that a highly sensitive alarm can be realized once the limit temperature is reached. Under higher temperature stimulation (>40 °C), there was no obvious trend in the numerical variation of *R*_C_ and it maintained at a high level, which was attributed to the strong correlation between *R*_C_ and thermal recovery deformation. Furthermore, the current response performance of RW‐TENG toward various airflow temperature stimulations under different rotating speeds (initial rotation frequency *f* = 10 Hz, 20 Hz) was investigated. As shown in Figure S12 (Supporting Information), the output current increased from ≈1 µA to ≈2 µA under hot wind stimulation at low rotating speed (10 Hz), and the output current increased from ≈3 to ≈6 µA with the hot wind stimulations under high rotating speed (20 Hz) (Figure S13, Supporting Information), showing approximate response values. However, the response time was longer than the low rotating speed (Figure S14, Supporting Information). The variation in response time may be attributed to the dissipative effect of heat generated by wind‐driven turbine generators during operation. As RW‐TENG has a more effective heat‐dissipating capacity at high rotation speeds, it takes a little longer to build up enough heat to regain SMA's shape when subjected to specific hot wind stimulation. It should be noted that RW‐TENG can work as an effective temperature alarm under varying wind or airflow rates, as the rotating speed has minimal effect on the response value.

Significantly, the increase in output current may also originate from the increased airflow speed of the cooler system. Therefore, it is necessary to distinguish whether higher airflow temperatures cause the response to eliminate the interference of wind speed. As shown in Figure 4f, frequency responses of the RW‐TENG were recorded toward 40–70 °C airflow, demonstrating a modest downward trend overall (the upward trend during the first 0 –10 s was ascribed to the thermionic emission effect). An initial current of 2 µA and 15 Hz was observed (*t* = 10 s, Figure 4f(i)), which came to 4 µA and 10 Hz after stabilization (*t* = 120 s, Figure 4f(ii)). The decrease in frequency was attributed to the greater electrostatic torque with the larger triboelectric area that hindered the rotation (Figure S15, Supporting Information). Detailly, it can be ascribed to the electrostatic attraction generated by the increased tribo‐charges, especially the substantial positive charges stored by the rabbit fur due to the fluffy structure, subjecting RW‐TENG to a larger electrostatic torque *M*_e_.^[^ ^59^ ^]^ Therefore, FEP is attracted by the positive charges on rabbit fur, which creates small gaps in the electrode. The generated air gaps between FEP and Cu electrodes decrease the electrostatic induction efficiency, resulting in decreased output. In contrast, the current frequency and amplitude rose simultaneously as the wind speed increased (Figure 3i). Therefore, further detection of the electrical output frequency can assist in boosting wind temperature‐selective performance.

**Figure 5:**
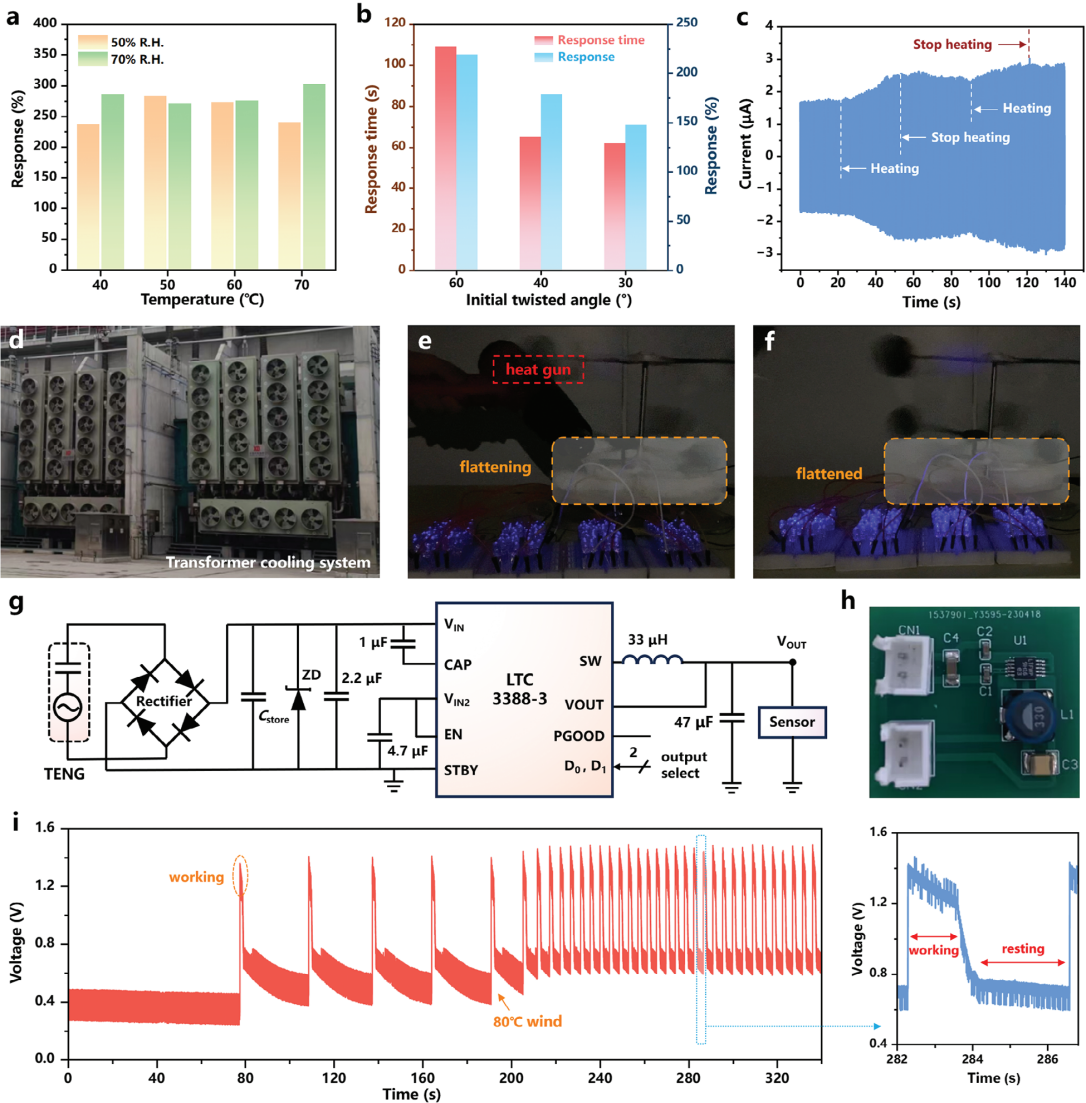
Application of the RW‐TENG for airflow temperature sensing in oil‐immersed transformers. a) Comparison of the RW‐TENG current response time under different relative humidity. b) Current response and response time of the RW‐TENG with various initial twisted angles (*α*_0_). c) Current response of RW‐TENG with periodic 40 °C hot wind stimulation. d) Photo demonstration of cooling‐fan arrays equipped by an air‐cooling transformer with forced oil circulation. RW‐TENG‐based self‐powered wind temperature alarm system in e) “flattening” state and f) “flattened” state. g) Circuit diagram of the RW‐TENG powering the commercial thermometer. h) The fabricated PCB for power management of RW‐TENG. i) The typical working waveform of the thermometer.

Furthermore, environmental relative humidity (R.H.) is an essential impact factor for RW‐TENG operation. Excessive ambient humidity will lead to the formation of a “water bridge” on the polymer surface, which shields the tribo‐charges and impairs the triboelectric performance.^[^ ^60^, ^61^ ^]^ However, water drops will evaporate exposed to hot airflow stimulation, which may as well lead to an output increase. To further investigate the dominant factor of the output, we first evaluated the triboelectric performance of the RW‐TENG under different R.H. conditions. As shown in Figure S16 (Supporting Information), the triboelectric outputs of RW‐TENG showed a noticeable decreasing trend as R.H increased from 30–70%. Specifically, the device maintained 27% output voltage and 31% current under 70% R.H. condition. However, there was no apparent change in triboelectric output performance and the rotator area after hot airflow stimulation, according to the response waveforms with SMA of “flattened” state initially upon hot airflow exposure under various R.H. conditions provided in Figure S17 (Supporting Information). The output current and voltage demonstrated a slight increase (20–25%) after hot airflow stimulation at 50% and 70% R.H., which could be attributed to the evaporation of water molecules captured by the rabbit fur. The results indicated that the current response under a high‐humidity environment is partially attributed to the drying of rabbit fur, while the contribution is insignificant compared to the ≈100% current increase illustrated in Figure 4d. Furthermore, current response waveforms of our RW‐TENG upon hot airflow exposure under 50% and 70% R.H. conditions were provided in **Figure** **5**a and Figures S18 and S19 (Supporting Information). The current response value slightly increased with the R.H., which may be attributed to the synergistic effect of the drying of rabbit fur as well as the increasing rotator contact area. To further prove our hypothesis, a response test was conducted (40 °C airflow) by reducing the initially twisted angle *α*_0_ (namely reducing the rotator area increment). It demonstrated a decreasing trend in both the response and the response time, as shown in Figure 5b and Figure S20 (Supporting Information), with *R*_C_ reaching only 48% within 62 s when *α*_0_ was reduced to 30 °. Therefore, we can infer that although high R.H. may increase the current response, the tunable rotator area was still primarily responsible for the wind temperature response.

Considering industrial equipment faults such as overheating may not be constant, a periodic hot wind stimulation strategy on RW‐TENG was employed (40 and 60 °C, respectively). According to the current response illustrated in Figure 5c and Figure S21 (Supporting Information), *I*_sc_ increased from 1.73 to 2.51 µA with a heating time of 31.6 s, and then slightly decreased to 2.38 µA during the interval 35.3 s, and finally increased to 3.02 µA with another heating time of 32.2 s for the 40 °C periodic wind stimulation. The situation for 60 °C periodic wind stimulation was similar, with the final current response value reaching 174.6% (40 °C) and 178.1% (60 °C), respectively. It indicated that the RW‐TENG response would be superimposed on the increased current amplitude resulting from the preceding overheat fault, and the alarm system would finally be triggered once enough response time has elapsed. The stability test for RW‐TENG with the SMA of “curved” state without hot wind simulation (Figure S22, Supporting Information) as well as RW‐TENG with the SMA of “flattened” state with 50 °C wind simulation (Figure S23, Supporting Information) was also conducted. The RW‐TENG can maintain the output current of ≈2 and ≈6 µA, respectively. The repeatability of the tunable area rotator was also investigated for five cycles, as shown in Figure S24 (Supporting Information). The response and response time maintained unchanged throughout each cycle, manifesting the enormous repeatability and durability of the device. Therefore, our suggested temperature monitoring system can satisfy the requirements of industrial applications.

To demonstrate the universal applicability of our device, shape memory NiTi alloy with *T*_g_ ≈50 °C was also employed to demonstrate the effect of *T*_g_ on the wind temperature monitoring device (the characteristics of SMA are shown in Figure S25, Supporting Information). According to Figure S26 (Supporting Information), the output showed no response toward 45 °C airflow as the NiTi alloy remained in the martensite phase of orthogonal or monoclinic crystal at a temperature lower than *T*_g_. Similar to the current response in Figure 4d, a remarkable current response was observed toward >60 °C airflow (Figure S26b–d, Supporting Information). The response value reached 160.8%, 161.9% and 178.9%, corresponding to 60, 65, and 70 °C airflow, respectively, as illustrated in Figure S27 (Supporting Information). However, RW‐TENG must absorb more heat from the outside hot airflow for a longer time due to the higher phase transition temperature to achieve shape recovery, which caused an increase in response time (149.7, 139.8, and 134.2 s corresponding to 60, 65, and 70 °C airflow, respectively). Consequently, when the required wind temperature to be alerting is too high, selecting SMA with a higher phase transition temperature can also be accomplished with some sacrifice in response time.

Figure 5d displays the real image of cooling‐fan arrays equipped by an air‐cooling transformer with forced oil circulation, indicating broad deployment prospects of the designed RW‐TENG in unmanned substations. The working principle of the oil‐immersed transformer cooling system was illustrated in Note S3 (Supporting Information). RW‐TENG can deliver timely alarm information to substation monitoring camera in the event of faults such as excessive air flow temperature in the cooler, which lowers the expense of on‐site operation and maintenance. As a proof of concept, the self‐powered airflow temperature alarm system was established. The RW‐TENG demonstrated the “curved” state as the transformer cooler operated under normal temperature (Figure S28, Supporting Information), and the alarm indicator composed of 200 series LEDs was not illuminated. When the hot air gun started heating (>40 °C), the RW‐TENG demonstrated the transient “flattening” state with the output voltage exceeding the LEDs threshold voltage, and the alarm indicator was weakly illuminated (Figure 5e). Once heated to the “flattened” state, the increased output current delivered a higher brightness for the alarm indicator, alerting dispatchers to reduce power load (Figure 5f). A more detailed demonstration can be found in Movie S1 (Supporting Information).

In addition, the RW‐TENG was connected to a high‐efficiency step‐down DC/DC converter (LTC3388‐3) after rectification to power the commercial thermometer (Figure 5g,h), and the typical working waveform is plotted in Figure 5i. Considering the integrated under voltage‐lockout (UVLO) strategy, LTC3388‐3 would disable the converter and maintain a low quiescent current state when the charging voltage of RW‐TENG to *C*_store_ is lower than 2.3 V. It would provide a standard DC output until the thermometer depletes the energy once the turn‐on voltage of the converter is reached. Finally, the printed circuit board (PCB) for power management was fabricated (Figure 5h) according to the above working principle, and the energy storage module and load sensors were connected to its ends. The thermometer could be lit up for 3.3 s (*V*_out_ > 1.4 V) by the RW‐TENG with the “curved” state, but it may take up to 30 s of dormancy for *C*_store_ to build up enough charges to turn on the DC/DC converter. The working/resting time ratio of the thermometer rapidly increased from 0.11 to 0.45 (the working period of 1.3 s and the resting period of 2.9 s) when the ambient wind temperature surpassed 40 °C, delivering the temperature information within a shorter amount of time until the arrival of maintenance personnel. A more intuitive demonstration can be found in Movie S2 (Supporting Information). Noticeably, due to the unidirectional SME of the NiTi alloy, it is necessary to mechanically deform the rotator sector before proceeding to the next stage of use when the cooler is functioning normally after troubleshooting. The comparison of detection limit between our proposed self‐powered wind monitoring device and commercial wind sensor is also demonstrated in Note S4 (Supporting Information). To be brief, the fabricated RW‐TENG could form a susceptible self‐powered active alarm system with promising application in real‐time airflow temperature detection in various industrial air cooling equipment.

## 3 Conclusion

In summary, we designed a soft‐contact mode RW‐TENG with a tunable rotator area via the thermal‐response shaping of the shape memory NiTi alloy. The device demonstrated promising application as the power source with an average power density of 140 mW m^−2^ at the wind speed of 12 m s^−1^, as a wind speed sensor with a sensitivity of 0.526 µA m^−1^ s at 2.2–19.6 m s^−1^. The thermal‐deformed NiTi alloy delivers a self‐adaptive contact area of the TENG dependent on the air temperature. Therefore, the RW‐TENG can serve as a self‐powered monitoring device of high airflow temperature, which show relatively short response time (109 s), strong anti‐interference ability and outstanding long‐term durability. This work introduces an innovative route for real‐time airflow and wind temperature detection in air‐cooled industrial equipment, promising for information perception and intelligent operation for the industrial Internet of Things.

## 4 Experimental Section

### Materials

Cu foil (thickness: 50 µm), FEP film (thickness: 100 µm), absolute alcohol and deionized water (DI) was purchased from Wuhan Xinshenshi Chemical Technology Co., Ltd. Shape memory NiTi alloy (0.5 mm) was purchased from Suzhou Chuanmao Co., Ltd. Rabbit fur (20 mm) was purchased from the open market.

### Fabrication of the Stator

An acrylic plate with Cu foil pasted on the surface was cut into a disc (outer diameter of 18 cm) using a laser cutting machine (JSLC‐1309‐w130) to form the substrate, and grooves were ground on the disc surface as illustrated in Figure 1b(ii). FEP film was polished by 320 mesh sandpaper and thoroughly cleaned with absolute alcohol and DI. FEP film was then attached to the surface of Cu electrodes.

### Fabrication of the Rotator

PLA sector supporting layer (outer diameter of 10 cm) was made by 3D printer (Tiretime, UP300). A small column with a hole was constructed into the sector substrate's center, allowing the air gap to be adjusted. NiTi alloy was cut into four sectors by a laser cutting machine, then pasted beneath the supporting layer. The trimmed rabbit fur was cut into the same shape as NiTi alloy, then cleaned with absolute alcohol and DI in turn. After drying, it was pasted beneath the NiTi alloy as the tribo‐positive layer.

### Assembly of the RW‐TENG

A ball bearing was placed into the inner circle of the stator substrate. An aluminum alloy shaft (diameter of 6 mm) was inserted into the rotator hole and the stator bearing from top to bottom of the RW‐TENG. The wind cups were all made by 3D printer, with its central sleeve nested on the top of the rotating shaft. The wire was always connected to the Cu electrode terminals in parallel during the experiment.

### Characterization and Measurement

A centrifugal blower (L‐CZR, 200 W) was used to simulate airflow. The industrial digital anemometer (TA8161) was used to measure the wind speed of the blower output. A hot wind gun (HG6618S) was used to simulate hot airflow. An industrial digital thermometer (TM902C) was employed to measure the wind temperature. The morphology of FEP and NiTi SMA were revealed by field‐emission SEM (FEI Inspect F50). The element distribution was explored by an energy dispersive spectrometer (EDS, EDAX OCTANE SUPERTA). The shape memory effect of NiTi SMA was determined using a differential scanning calorimeter (DSC, TA DSC25). The surface roughness was explored using a tapping‐mode atomic force microscope (AFM, Dimension ICON). Temperature distribution images of RW‐TENG were obtained using an infrared thermal imaging camera (Guide C‐640). The electrometer (6517B, Keithley) connected with the multimeter (DAQ 6510, Keithley) was utilized to measure the short‐circuit current, open‐circuit voltage, and transferred charge of the RW‐TENG, and a personal computer with TPS‐4000 software recorded the acquired data.

### Calculation Method

The potential difference distribution is simulated by the AC/DC module of COMSOL Multiphysics 5.5. The surface charge densities of rabbit fur and FEP were assumed to be 1 µC m^−2^ and −500 nC m^−2^, respectively. The recovery ratio (*R*) of the SMA is expressed as:

(3) $$R\;\left(\%\right)\;=\left(\alpha_{0}-\alpha \left(t\right)\right)/\alpha_{0}\;\times \;100\%$$

where *α*(*t*) is the angle of the twisted rotator material during the recovery.

To quantitatively evaluate the impact of the airflow temperature on RW‐TENG performance, the response (*R*_C_) was defined as the relative current change upon hot airflow compared to the stable output current, which is calculated as follows:

(4) $$R_{C}\left(\%\right)\;=I_{h}/I_{0}\;\times \;100\%$$

where *I*_0_ referred to the initial RW‐TENG current in cool airflow and *I*_h_ referred to the RW‐TENG current in target hot airflow.

### Statistical Analysis

1. Pre‐processing of data: uninvolved.
2. Data presentation: in Figure 2a,b,c, Figure S8 (Supporting Information), the short‐circuit current is presented with the mean plus and the error bar. In Figures 2f and 3d,e,i, Figure S6 (Supporting Information), the data is presented with the mean plus.
3. Sample size (n) for each statistical analysis: in Figures 2a,b,c,f, and 3d,e,i, Figures S6 and S8 (Supporting Information), data were taken from the average of 20 data points of steady‐state peak value, and data points in the figure are taken from the average of three average peak value points.
4. Statistical methods used to assess significant differences with sufficient details: uninvolved.
5. Software used for statistical analysis: MATLAB 2023a and OriginLab 2023b.

## Conflict of Interest

The authors declare no conflict of interest.

## Supporting information

Supporting Information

Supplemental Movie 1

Supplemental Movie 2

## Data Availability

The data that support the findings of this study are available from the corresponding author upon reasonable request.

## References

[1] L. Paulhiac , R. Desquiens , IEEE Trans. Power Delivery 2022, 37, 4135.

[2] N. Acharya , Int. Commun. Heat and Mass Transfer 2022, 133, 105980.

[3] N. Acharya , A. J. Chamkha , Int. Commun. in Heat and Mass Transfer 2022, 132, 105885.

[4] F. Chen , R. Huang , C. Wang , X. Yu , H. Liu , Q. Wu , K. Qian , R. Bhagat , Appl. Therm. Eng. 2020, 173, 115154.

[5] H. Behi , D. Karimi , M. Behi , M. Ghanbarpour , J. Jaguemont , M. A. Sokkeh , F. H. Gandoman , M. Berecibar , J. Van Mierlo , Appl. Therm. Eng. 2020, 174, 115280.

[6] J. Lopez , J. E. Rubio , C. Alcaraz , IEEE Wireless Commun. 2021, 28, 48.

[7] H. Chen , X. Wang , Z. Li , W. Chen , Y. Cai , Prot. Control Mod. Power Syst. 2019, 4, 13.

[8] A. A. Shobole , M. Wadi , Renewable Sustainable Energy Rev. 2021, 149, 111352.

[9] G. Bedi , G. K. Venayagamoorthy , R. Singh , R. R. Brooks , K.‐C. Wang , IEEE Internet Things J. 2018, 5, 847.

[10] C. Jiang , X. Li , S. W. M. Lian , Y. Ying , J. S. Ho , J. Ping , ACS Nano 2021, 15, 9328. 34124880 10.1021/acsnano.1c02819

[11] P. Cui , Y. Ge , X. Yao , J. Wang , J. Zhang , H. Meng , L. Liu , J. Wang , J. Ju , G. Cheng , Z. Du , Nano Energy 2023, 109, 108286.

[12] D. Choi , Y. Lee , Z.‐H. Lin , S. Cho , M. Kim , C. K. Ao , S. Soh , C. Sohn , C. K. Jeong , J. Lee , M. Lee , S. Lee , J. Ryu , P. Parashar , Y. Cho , J. Ahn , I.‐D. Kim , F. Jiang , P. S. Lee , G. Khandelwal , S.‐J. Kim , H. S. Kim , H.‐C. Song , M. Kim , J. Nah , W. Kim , H. G. Menge , Y. T. Park , W. Xu , J. Hao , et al., ACS Nano 2023, 17, 11087. 37219021 10.1021/acsnano.2c12458 PMC10312207

[13] L. Zhou , D. Liu , J. Wang , Z. L. Wang , Friction 2020, 8, 481.

[14] W.‐G. Kim , D.‐W. Kim , I.‐W. Tcho , J.‐K. Kim , M.‐S. Kim , Y.‐K. Choi , ACS Nano 2021, 15, 258. 33427457 10.1021/acsnano.0c09803

[15] H. Wen , X. Yang , R. Huang , D. Zheng , J. Yuan , H. Hong , J. Duan , Y. Zi , Q. Tang , Adv. Sci. 2023, 10, 2204694. 10.1002/advs.202302009 PMC10401095 37246274

[16] P. Wu , F. Wang , S. Xu , T. Liu , Y. Qi , X. Zhao , C. Zhang , X. Mu , Adv. Sci. 2023, 10, 2301199. 10.1002/advs.202301199 PMC10375136 37132585

[17] X. Tang , W. Hou , Q. Zheng , L. Fang , R. Zhu , L. Zheng , Nano Energy 2022, 99, 107412.

[18] Y.‐C. Lai , Y.‐C. Hsiao , H.‐M. Wu , Z. L. Wang , Adv. Sci. 2019, 6, 1801883. 10.1002/advs.201801883 PMC6402409 30886807

[19] M. Song , J. Hur , D. Heo , S.‐H. Chung , D. Kim , S. Kim , D. Kim , Z.‐H. Lin , J. Chung , S. Lee , Appl. Energy 2023, 344, 121248.

[20] L. He , C. Zhang , B. Zhang , O. Yang , W. Yuan , L. Zhou , Z. Zhao , Z. Wu , J. Wang , Z. L. Wang , ACS Nano 2022, 16, 6244. 35312283 10.1021/acsnano.1c11658

[21] Z. Ren , Z. Wang , Z. Liu , L. Wang , H. Guo , L. Li , S. Li , X. Chen , W. Tang , Z. L. Wang , Adv. Energy Mater. 2020, 10, 2001770.

[22] Y. Zhang , Q. Zeng , Y. Wu , J. Wu , S. Yuan , D. Tan , C. Hu , X. Wang , Nano‐Micro Lett. 2020, 12, 175. 10.1007/s40820-020-00513-2 PMC7770936 34138173

[23] H.‐J. Ko , D.‐S. Kwon , K. Bae , J. Kim , Nano Energy 2022, 96, 107062.

[24] M. Lian , J. Sun , D. Jiang , M. Xu , Z. Wu , B. Bin Xu , H. Algadi , M. Huang , Z. Guo , Nanotechnology 2023, 34, 025401. 10.1088/1361-6528/ac97f1 36306413

[25] J. Han , Y. Feng , P. Chen , X. Liang , H. Pang , T. Jiang , Z. L. Wang , Adv. Funct. Mater. 2022, 32, 2108580.

[26] A. C. Wang , B. Zhang , C. Xu , H. Zou , Z. Lin , Z. L. Wang , Adv. Funct. Mater. 2020, 30, 1909384.

[27] M. T. Rahman , S. M. S. Rana , P. Maharjan , M. Salauddin , T. Bhatta , H. Cho , C. Park , J. Y. Park , Nano Energy 2021, 85, 105974.

[28] P. Lu , H. Pang , J. Ren , Y. Feng , J. An , X. Liang , T. Jiang , Z. L. Wang , Adv. Materi. Technol. 2021, 6, 2100496.

[29] Y. Pang , X. Zhu , Y. Jin , Z. Yang , S. Liu , L. Shen , X. Li , C. Lee , Appl. Energy 2023, 348, 121515.

[30] S. Shen , Y. Zhao , R. Cao , H. Wu , W. Zhang , Y. Zhu , K. Ren , C. Pan , Nano Energy 2023, 110, 108347.

[31] X. Tao , S. Li , Y. Shi , X. Wang , J. Tian , Z. Liu , P. Yang , X. Chen , Z. L. Wang , Adv. Funct. Mater. 2021, 31, 2106082.

[32] J. Wang , W. Ding , L. Pan , C. Wu , H. Yu , L. Yang , R. Liao , Z. L. Wang , ACS Nano 2018, 12, 3954. 29595963 10.1021/acsnano.8b01532

[33] S. Xiao , H. Wu , N. Li , X. Tan , H. Deng , X. Zhang , J. Tang , Y. Li , Adv. Sci. 2023, 10, 2207230. 10.1002/advs.202207230 PMC10161025 36825678

[34] Y. Wang , X. Yu , M. Yin , J. Wang , Q. Gao , Y. Yu , T. Cheng , Z. L. Wang , Nano Energy 2021, 82, 105740.

[35] A. Melocchi , M. Uboldi , M. Cerea , A. Foppoli , A. Maroni , S. Moutaharrik , L. Palugan , L. Zema , A. Gazzaniga , Adv. Drug Delivery Rev. 2021, 173, 216. 10.1016/j.addr.2021.03.013 33774118

[36] J. M. McCracken , B. R. Donovan , T. J. White , Adv. Mater. 2020, 32, 1906564. 10.1002/adma.201906564 32133704

[37] X. Niu , M. Wang , Y. Xia , Y. Zhu , X. Jia , R. Cao , X. Wang , ACS Appl. Mater. Interfaces 2022, 14, 50101. 10.1021/acsami.2c13294 36301079

[38] L.‐B. Huang , J.‐C. Han , S. Chen , Z. Sun , X. Dai , P. Ge , C.‐H. Zhao , Q.‐Q. Zheng , F.‐C. Sun , J. Hao , Nano Energy 2021, 84, 105873.

[39] J. Xiong , H. Luo , D. Gao , X. Zhou , P. Cui , G. Thangavel , K. Parida , P. S. Lee , Nano Energy 2019, 61, 584.

[40] G. Yang , H. Li , R. Xing , M. Lv , C. Ma , J. Yan , X. Zhuang , Adv. Funct. Materi. 2023, 33, 2214001.

[41] G. L. W. Hart , T. Mueller , C. Toher , S. Curtarolo , Nat. Rev. Mater. 2021, 6, 730.

[42] L. He , C. Zhang , B. Zhang , Y. Gao , W. Yuan , X. Li , L. Zhou , Z. Zhao , Z. L. Wang , J. Wang , Nano Energy 2023, 108, 108244.

[43] S. Yong , J. Wang , L. Yang , H. Wang , H. Luo , R. Liao , Z. L. Wang , Adv. Energy Mater. 2021, 11, 2101194.

[44] X. Zhu , X. Cao , Z. L. Wang , Adv. Mater. Technol. 2022, 7, 2200006.

[45] D. Lee , S. Cho , S. Jang , Y. Ra , Y. Jang , Y. Yun , D. Choi , Nano Energy 2022, 102, 107638.

[46] Z. Yuan , X. Jin , R. Li , B. Wang , C. Han , Y. Shi , Z. Wu , Z. L. Wang , Small 2022, 18, 2107221. 10.1002/smll.202107221 35678105

[47] X. Jin , Z. Yuan , Y. Shi , Y. Sun , R. Li , J. Chen , L. Wang , Z. Wu , Z. L. Wang , Adv. Funct. Mater. 2022, 32, 2108827.

[48] S. Gao , X. Zeng , G. Zhang , J. Zhang , Y. Chen , S. Feng , W. Lan , J. Zhou , Z. L. Wang , Nano Energy 2022, 101, 107530.

[49] S. Hu , Z. Yuan , R. Li , Z. Cao , H. Zhou , Z. Wu , Z. L. Wang , Nano Lett. 2022, 22, 5584. 35733084 10.1021/acs.nanolett.2c01912

[50] H. Wu , J. Wang , Z. Wu , S. Kang , X. Wei , H. Wang , H. Luo , L. Yang , R. Liao , Z. L. Wang , Adv. Energy Mater. 2022, 12, 2103654.

[51] C. He , T. Yang , J. Fang , X. Pu , K. Shang , G. Tian , X. Lu , J. Wu , W. Yang , L. Qian , Nano Energy 2023, 109, 108279.

[52] C. Men , X. Liu , Y. Chen , S. Liu , S. Wang , S. Gao , Nano Energy 2022, 101, 107578.

[53] R. Guo , K. Zhuo , Q. Li , T. Wang , S. Sang , H. Zhang , Appl. Energy 2023, 348, 121509.

[54] G. Zhu , J. Chen , T. Zhang , Q. Jing , Z. L. Wang , Nat. Commun. 2014, 5, 3426. 24594501 10.1038/ncomms4426

[55] X. Li , Y. Cao , X. Yu , Y. Xu , Y. Yang , S. Liu , T. Cheng , Z. L. Wang , Appl. Energy 2022, 306, 117977.

[56] S. Yong , H. Wang , Z. Lin , X. Li , B. Zhu , L. Yang , W. Ding , R. Liao , J. Wang , Z. L. Wang , Adv. Energy Mater. 2022, 12, 2202469.

[57] R. Xia , R. Zhang , Y. Jie , W. Zhao , X. Cao , Z. Wang , Nano Energy 2022, 92, 106685.

[58] C. Xu , A. C. Wang , H. Zou , B. Zhang , C. Zhang , Y. Zi , L. Pan , P. Wang , P. Feng , Z. Lin , Z. L. Wang , Adv. Mater. 2018, 30, 1803968. 10.1002/adma.201803968 30091484

[59] D. Liu , C. Li , P. Chen , X. Zhao , W. Tang , Z. L. Wang , Adv. Energy Mater. 2023, 13, 2202691.

[60] K. Wang , Z. Qiu , J. Wang , Y. Liu , R. Chen , H. An , J. H. Park , C. H. Suk , C. Wu , J. Lin , T. W. Kim , Nano Energy 2022, 93, 106880.

[61] R. Wen , J. Guo , A. Yu , J. Zhai , Z. L. Wang , Adv. Funct. Mater. 2019, 29, 1807655.

