# Supplementary material for: Rotary Wind‐driven Triboelectric Nanogenerator for Self‐Powered Airflow Temperature Monitoring of Industrial Equipment
Source: Adv Sci (Weinh). 2024 Jan 19;11(13):2307382. doi: 10.1002/advs.202307382 (PMC10987131; doi:10.1002/advs.202307382)
Supplement: Supplementary file 1 — Supporting Information [file ADVS-11-2307382-s001.pdf]

ADVANCED SCIENCE
Open Access

Supporting Information

for *Adv. Sci.*, DOI 10.1002/advs.202307382

Rotary Wind-driven Triboelectric Nanogenerator for Self-Powered Airflow Temperature Monitoring of Industrial Equipment

Yi Li, Haocheng Deng, Haoying Wu, Yi Luo, Yeqiang Deng, Hongye Yuan, Zhaolun Cui, Ju Tang, Jiaqing Xiong, Xiaoxing Zhang* and Song Xiao**

Supporting Information

Rotary Wind-driven Triboelectric Nanogenerator for Self-Powered Airflow Temperature Monitoring of Industrial Equipment

Yi Li [#], Haocheng Deng [#], Haoying Wu, Yi Luo, Yeqiang Deng, Hongye Yuan, Zhaolun Cui, Ju Tang, Jiaqing Xiong[*], Xiaoxing Zhang[*], Song Xiao[*]

Dr. Yi Li, Mr. H. C. Deng, Mr. H. Y. Wu, Dr. Y.Q. Deng, Prof. J. Tang, Prof. S. Xiao
State Key Laboratory of Power Grid Environmental Protection, School of Electrical Engineering and Automation, Wuhan University, Wuhan, Hubei 430072, China.
Dr. Y. Luo
Beijing International S&T Cooperation Base for Plasma Science and Energy Conversion, Institute of Electrical Engineering, Chinese Academy of Sciences, Beijing 100190, China.
Prof. H. Y. Yuan
State Key Laboratory for Mechanical Behavior of Materials, Shaanxi International Research Center for Soft Matter, School of Materials Science and Engineering, Xi'an Jiaotong University, Xi'an, 710049, China
Dr. Z. L. Cui
School of Electrical Power, South China University of Technology, Guangdong 510640, China.
Prof. J. Q. Xiong
Innovation Center for Textile Science and Technology, Donghua University, Shanghai 201620, China
Prof. X. X. Zhang
Hubei Engineering Research Center for Safety Monitoring of New Energy and Power Grid Equipment, Hubei University of Technology, Wuhan, Hubei 430068, China.

[#]The authors contribute equally

[*]Corresponding author Email: xiaoxing.zhang@outlook.com, xiaosong@whu.edu.cn, jqxiong@dhu.edu.cn

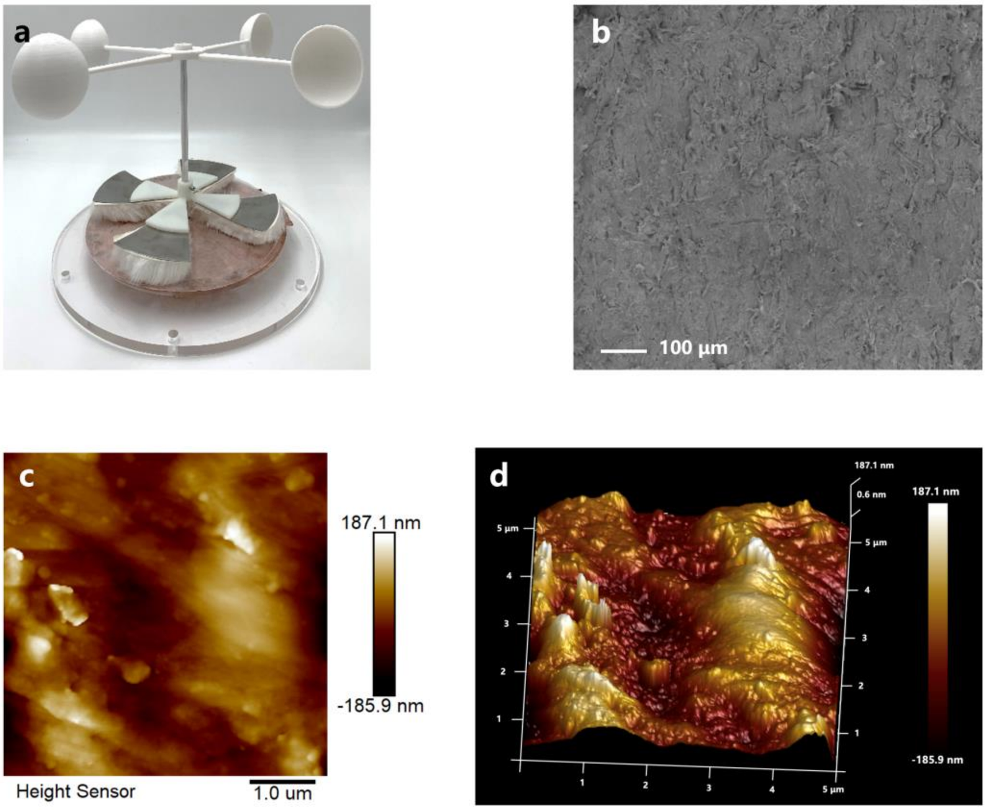

Figure S1. (a) Photograph of the as-fabricated RW-TENG. (b) SEM morphology of the FEP polished by 320 mesh sandpaper. (c-d) AFM images of the 320 mesh sandpaper polished FEP film (R_q = 53.2 nm, R_a = 42.4 nm).

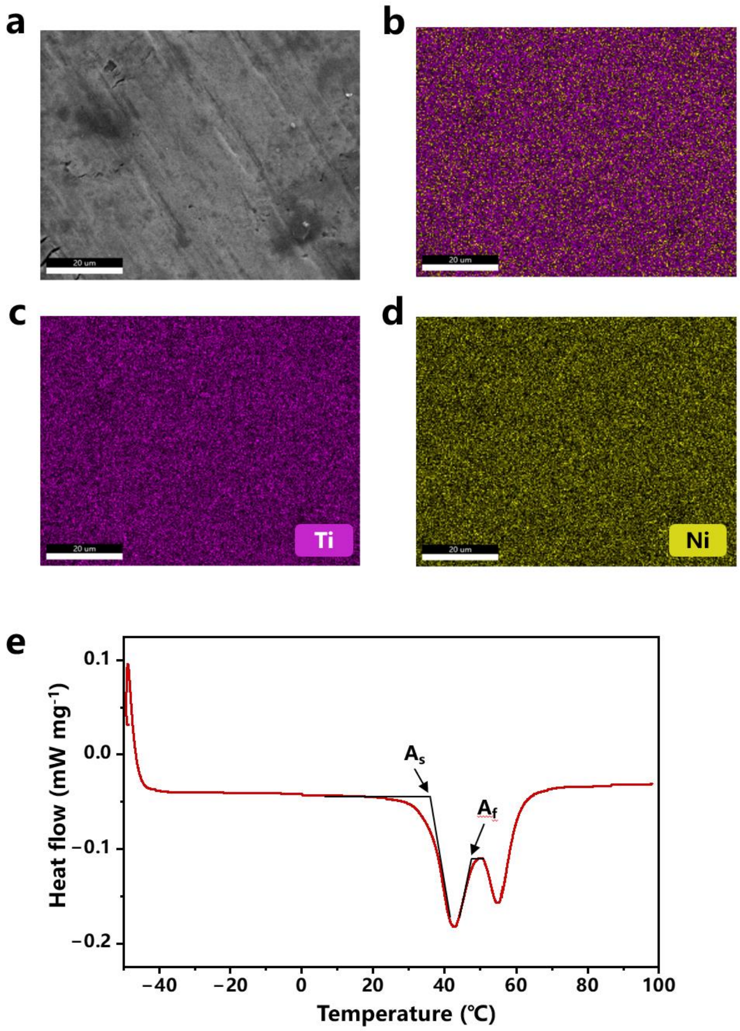

Figure S2. (a) Surface morphology of the shape memory NiTi alloy (T_g ~ 40°C). (b-d) Element distribution of Ti and Ni. (e) DSC curves of the shape memory NiTi alloy (T_g ~ 40°C).

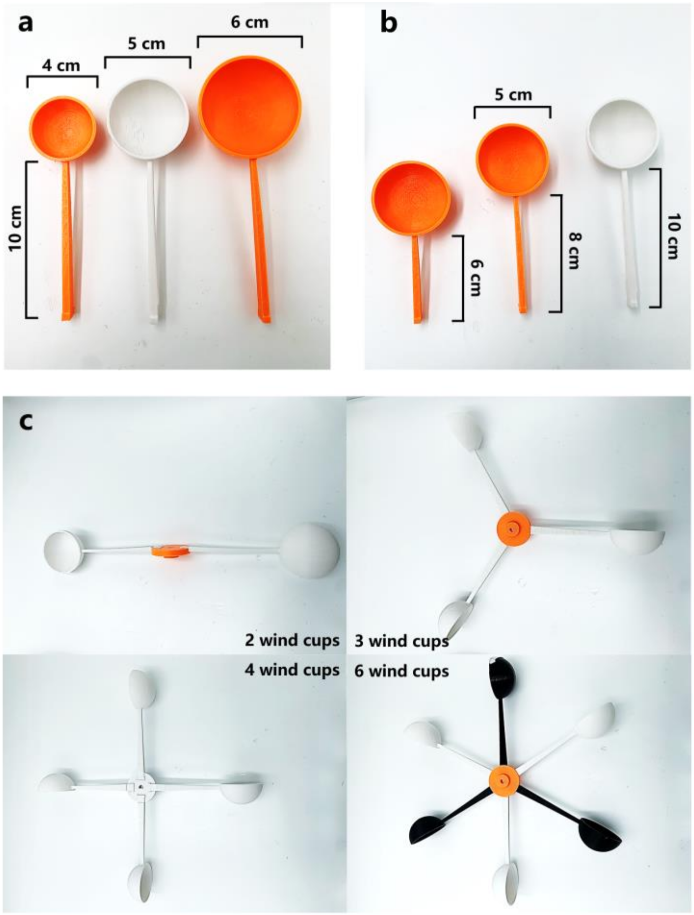

Figure S3. Photograph of the as-fabricated wind cups with various (a) diameters, (b) arm lengths, and (c) numbers.

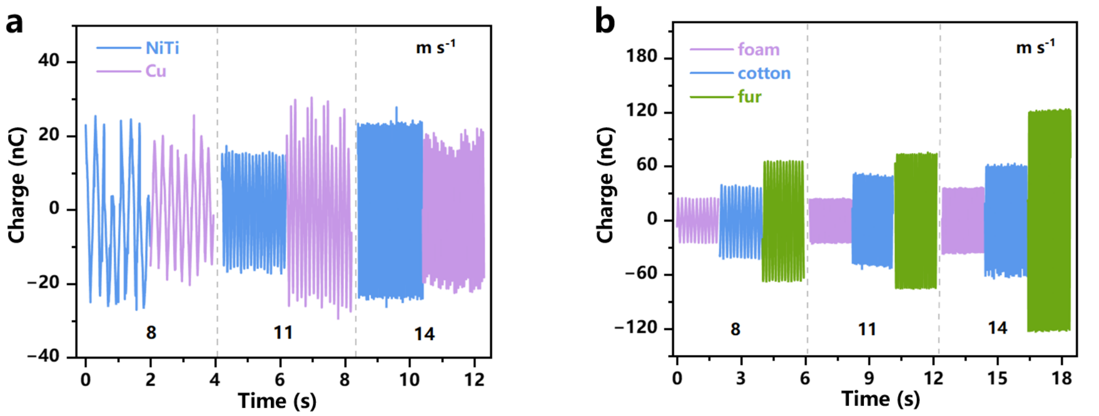

Figure S4. The transferred charge of the RW-TENG in (a) direct-contact mode and (b) soft-contact mode.

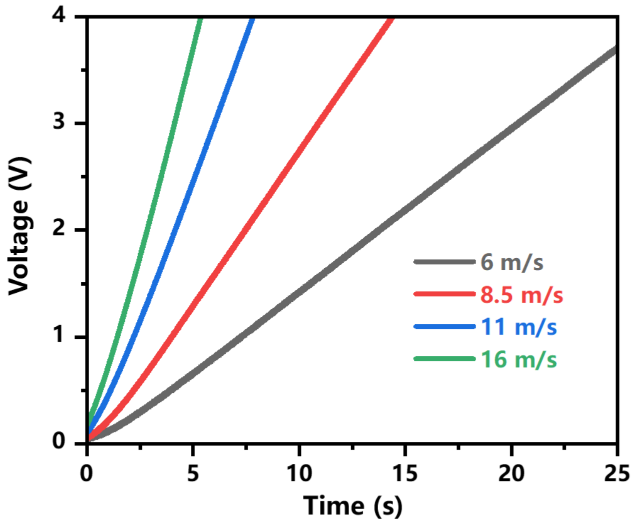

Figure S5. Charging curves of the capacitor (0.1 μF) by the RW-TENG under different wind speed conditions.

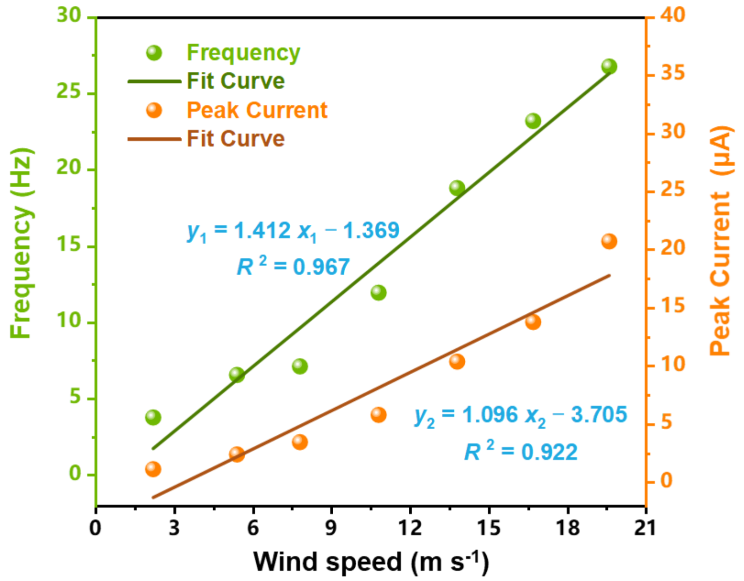

Figure S6. Measured relationship between the short-circuit current (peak value and frequency) and the wind speed under an air gap of 14 mm.

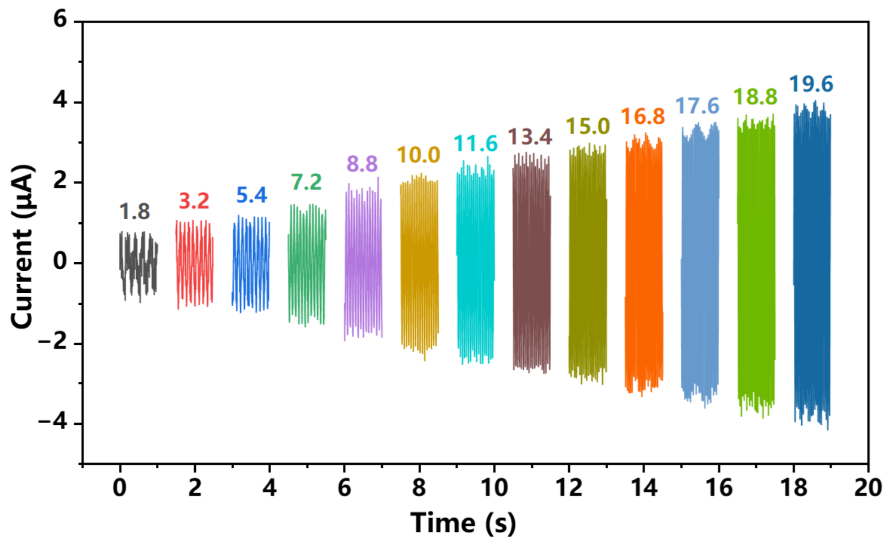

Figure S7. The short-circuit current of RW-TENG with curved SMA under various wind speeds.

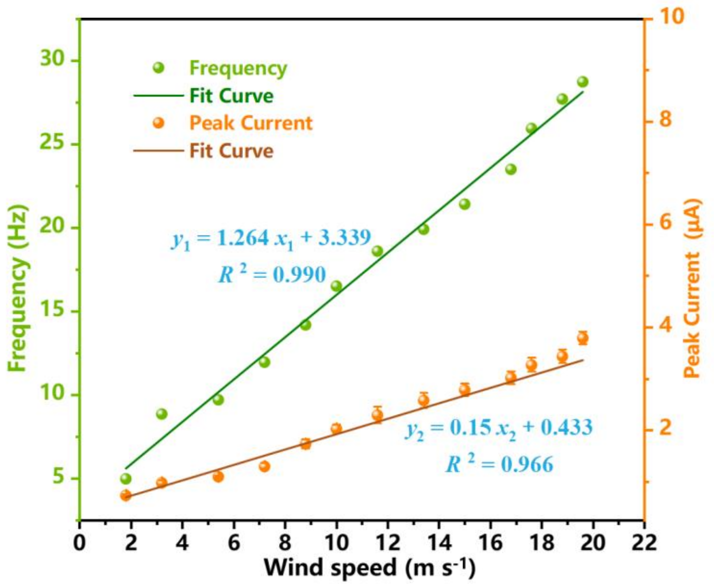

Figure S8. Measured relationship between the short-circuit current, frequency and wind speed of RW-TENG with curved SMA.

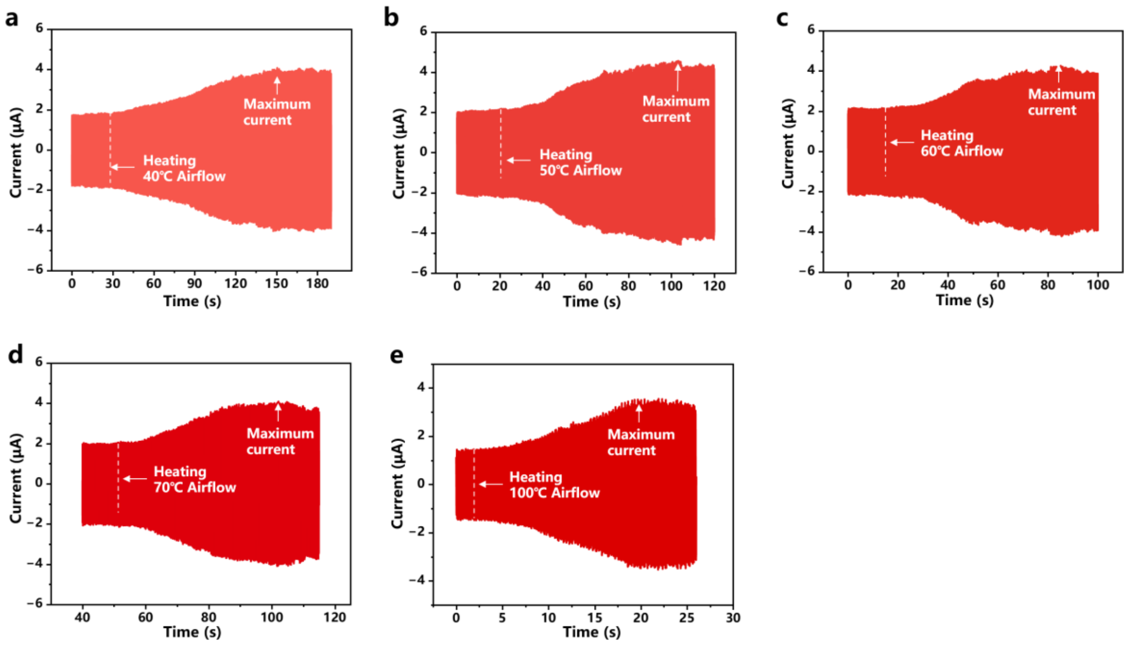

Figure S9. The entire current waveform of RW-TENG toward various (a) 40℃, (b) 50℃, (c) 60℃, (d) 70℃, and (e) 100℃ wind temperature stimulations.

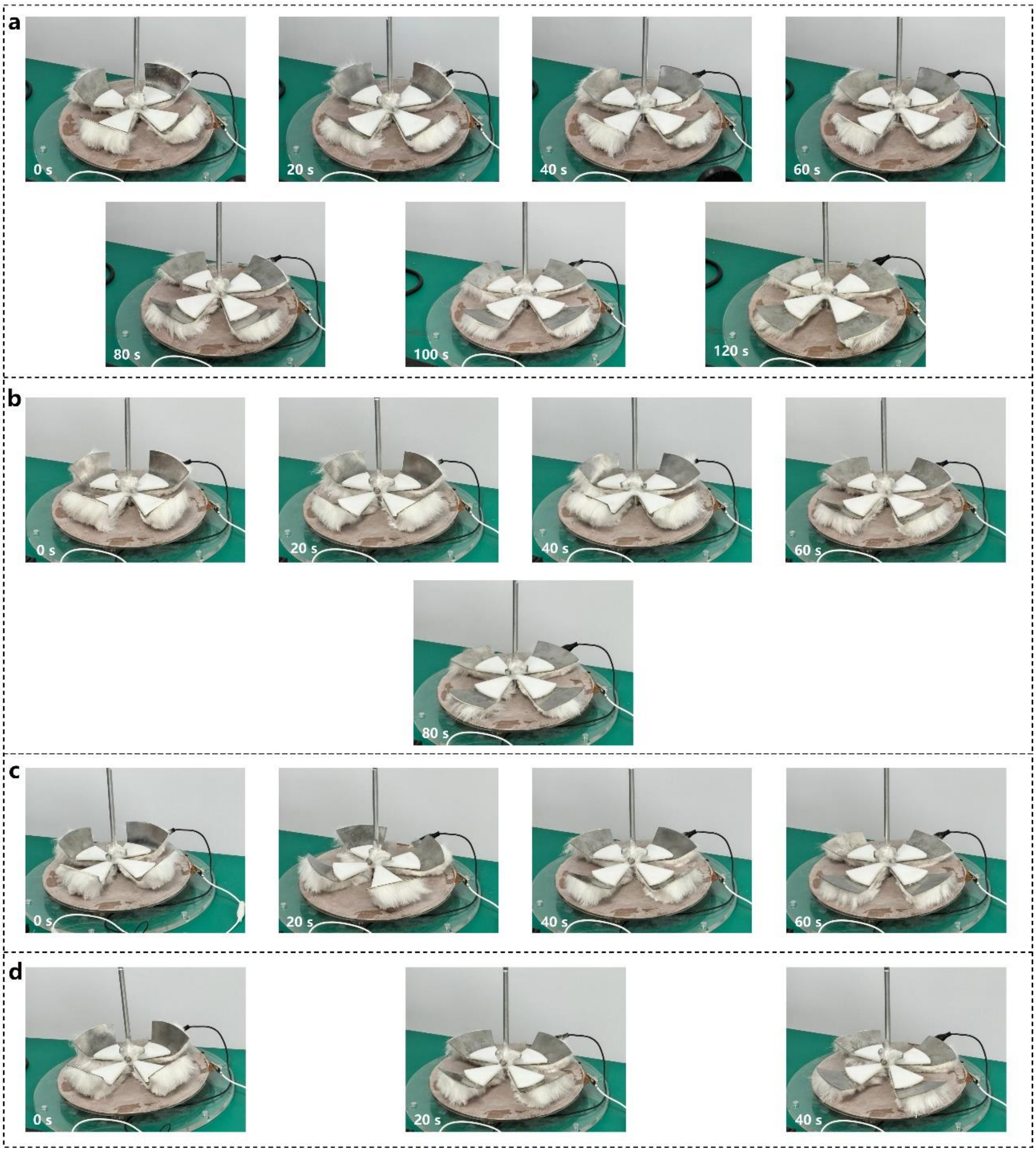

Figure S10. Physical image of the rotator of RW-TENG toward various stimulation temperatures. (a) 40°C, (b) 50°C, (c) 60°C, and (d) 70°C. The images were captured every 20 seconds until the shape of NiTi SMA no longer changed (test conditions: initial rotation frequency *f* = 15 Hz).

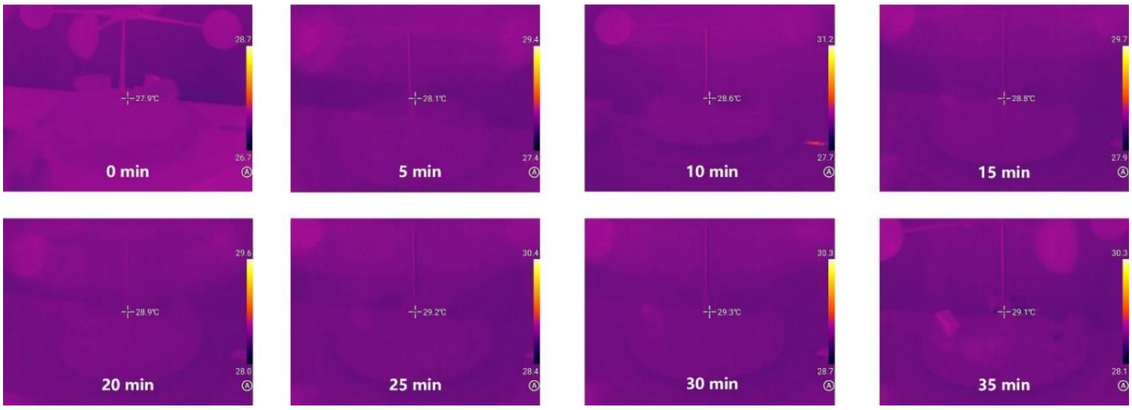

Figure S11. The temperature distribution images of RW-TENG during long-term operation (35 min) under the "curved" state.

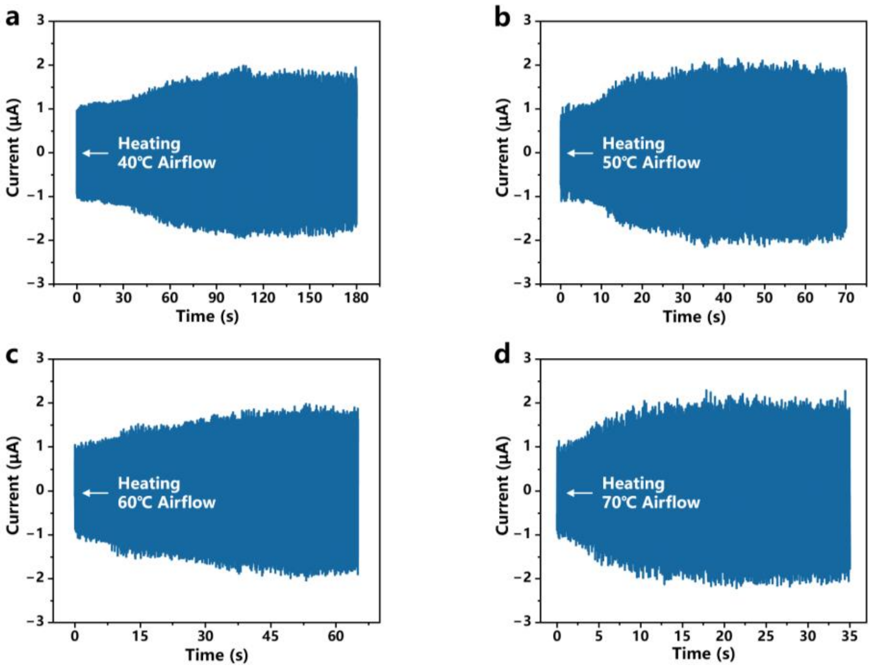

Figure S12. Current response waveform of RW-TENG toward various wind temperature stimulations under low rotating speed (initial rotation frequency f = 10 Hz).

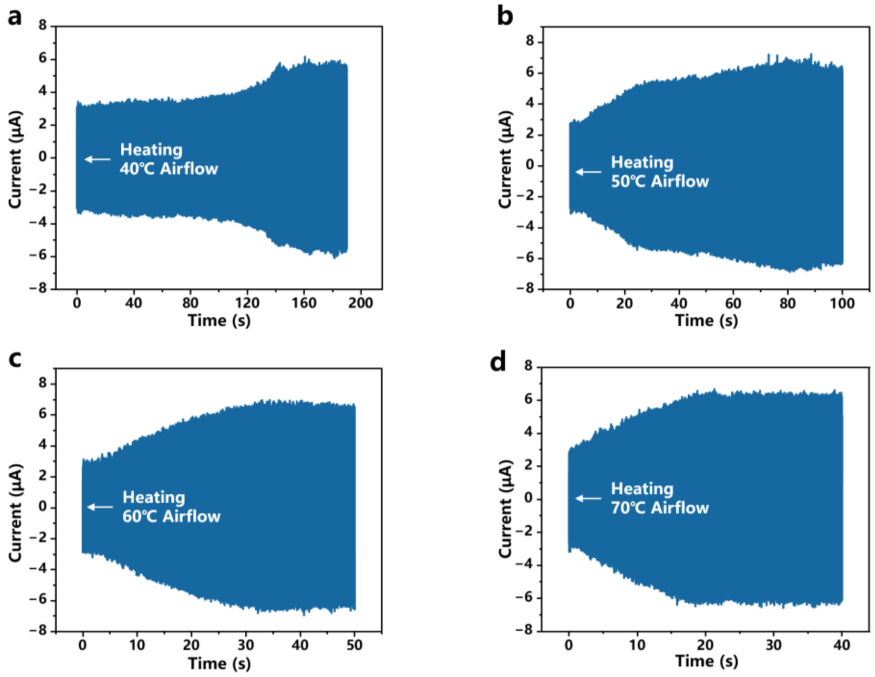

Figure S13. Current response waveform of RW-TENG toward various wind temperature stimulations under high rotating speed (initial rotation frequency $f = 20$ Hz).

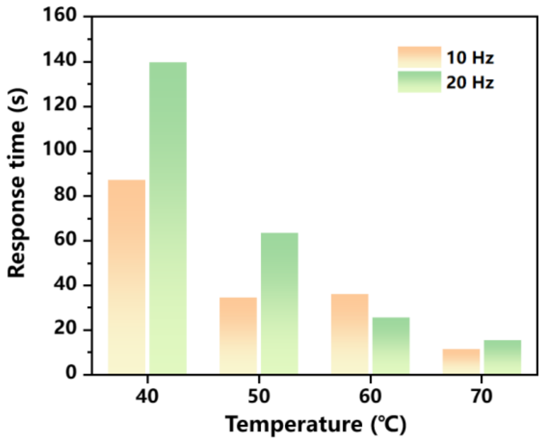

Figure S14. Comparison of RW-TENG current response time under different rotating speeds. The accurate current response time reached 87.1 s, 34.2 s, 36 s, and 11.4 s, respectively, corresponding to 40℃, 50℃, 60℃, and 70℃ airflow stimulations under low rotating speed (10 Hz). The current response value increased to139.7 s, 63.5 s, 25.4 s, and 15.5 s, respectively, under high rotating speed (20 Hz).

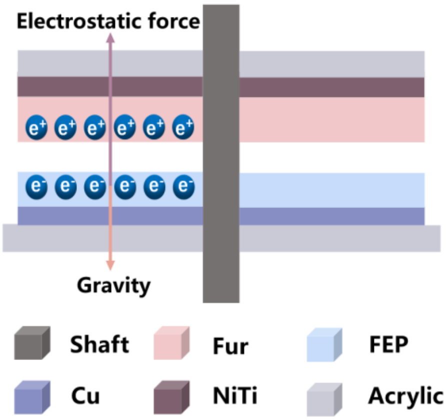

Figure S15. Force analysis of stator FEP during the operation of RW-TENG. As the rotator area increases, the stator FEP will be subjected to greater electrostatic attraction, therefore hindering the device rotation.

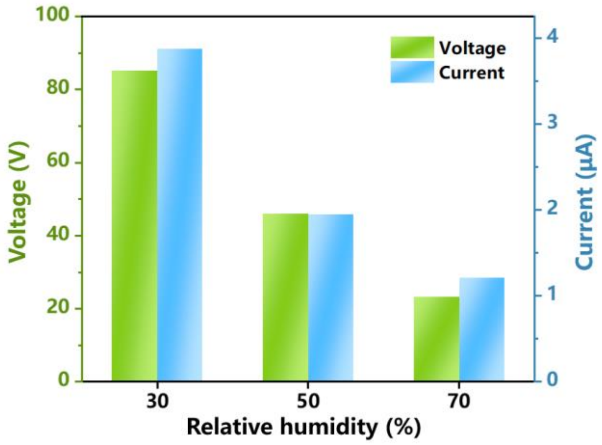

Figure S16. Triboelectric output voltage and current of RW-TENG under different humidity conditions (SMA of "flattened" state).

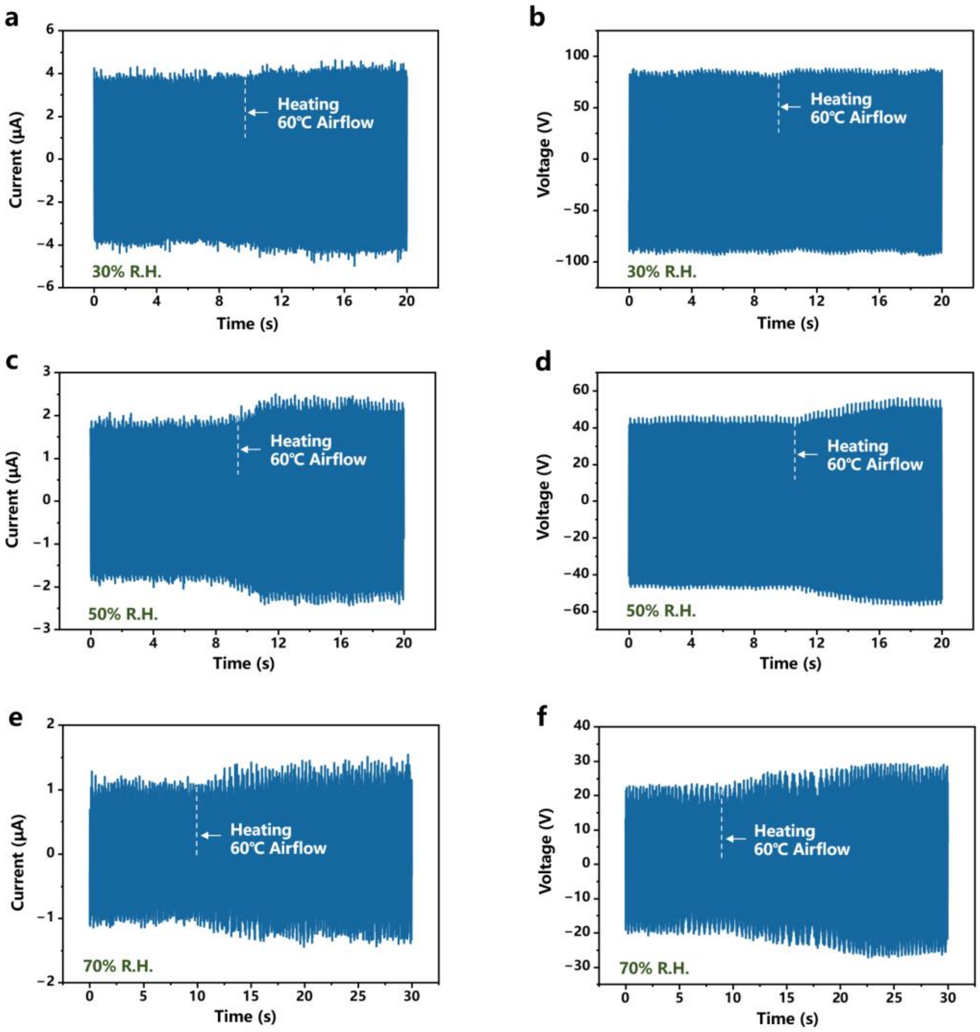

Figure S17. Output waveform of RW-TENG with SMA of "flattened" state upon 60℃ hot airflow exposure under (a-b) 30% R.H., (c-d) 50% R.H., and (e-f) 70% R.H.

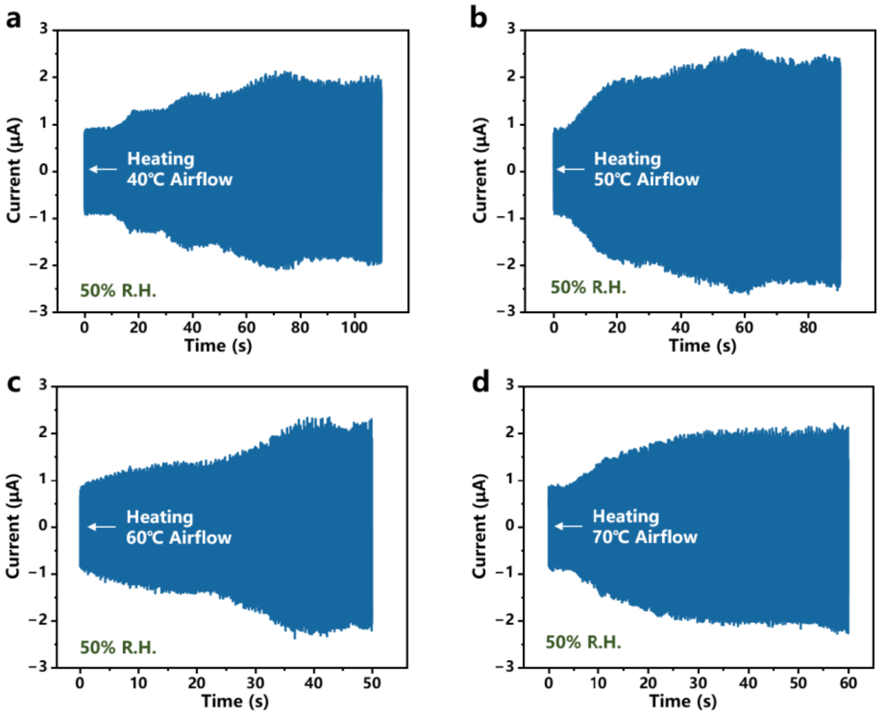

Figure S18. Current response waveform of RW-TENG toward various wind temperature stimulations when the environmental relative humidity is 50%.

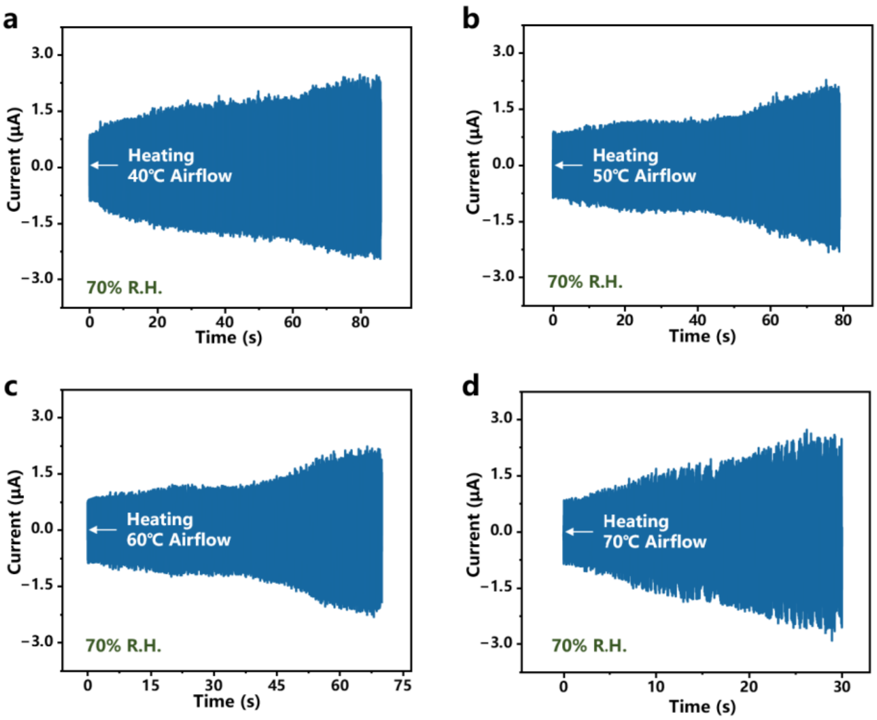

Figure S19. Current response waveform of RW-TENG toward various wind temperature stimulations when the environmental relative humidity is 70%.

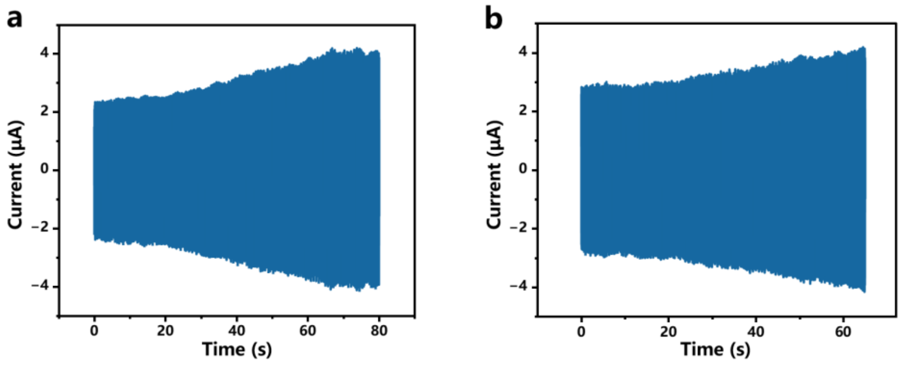

Figure S20. The current response waveform of RW-TENG toward 40 °C airflow stimulation with the twisted angle of (a) 40° and (b) 30°.

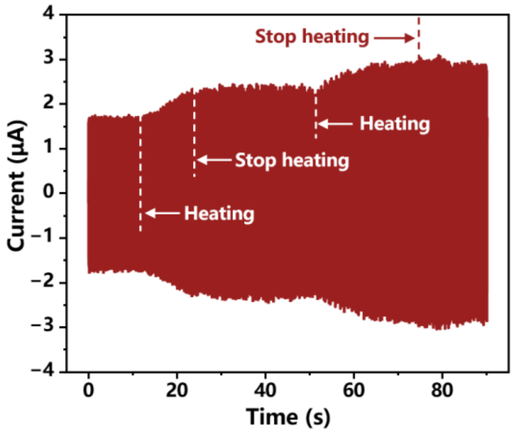

Figure S21. Current response of RW-TENG with periodic 60°C hot wind stimulation. The output short-circuit current increased from 1.69 μA to 2.34 μA with a heating time of 11.5 s, and then slightly decreased to 2.2 μA with an interval of 28.1 s, and then increased to 3.01 μA with another heating time of 23.4 s.

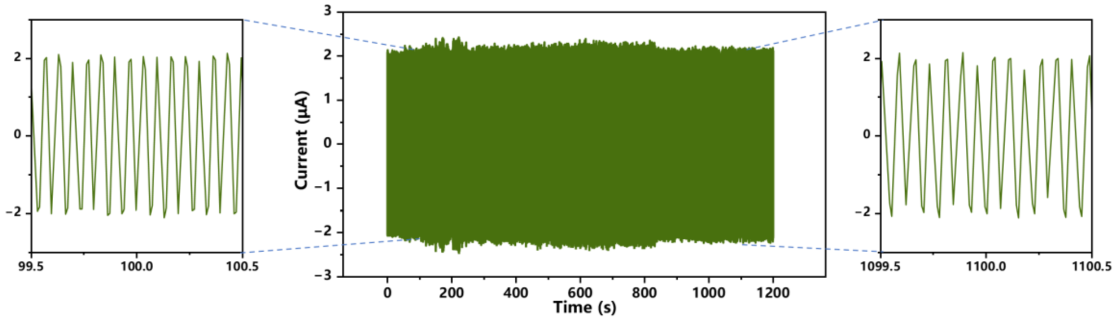

Figure S22. The output durability of RW-TENG with the SMA of "curved" state without hot wind simulation under continuous measurement of 1200 s.

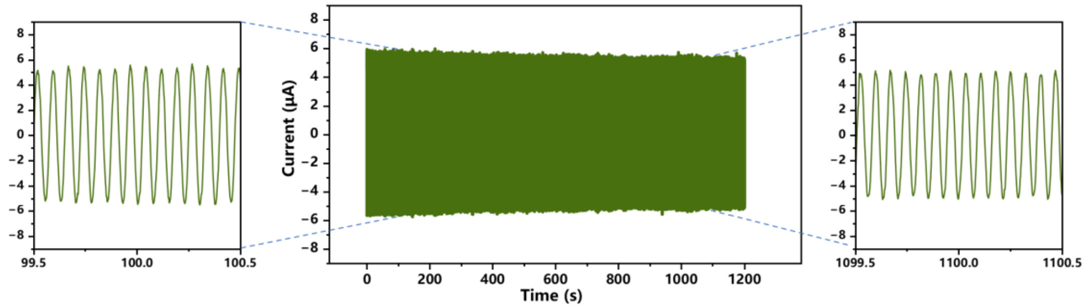

Figure S23. The output durability of RW-TENG with the SMA of "flattened" state with 50°C wind simulation under continuous measurement of 1200 s.

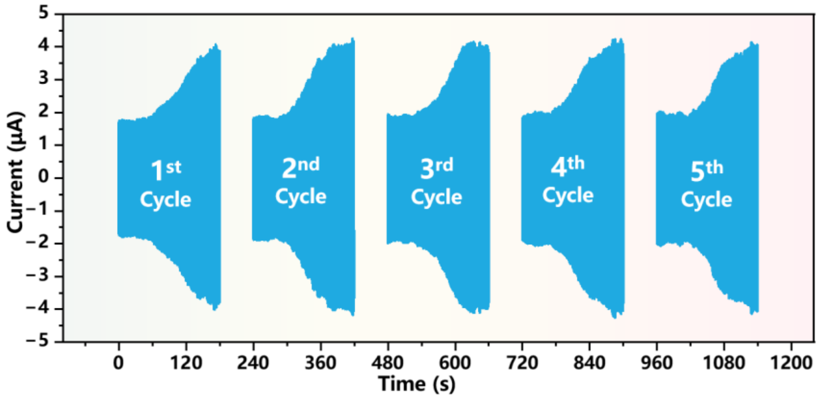

Figure S24. Response repeatability of the RW-TENG for five cycles.

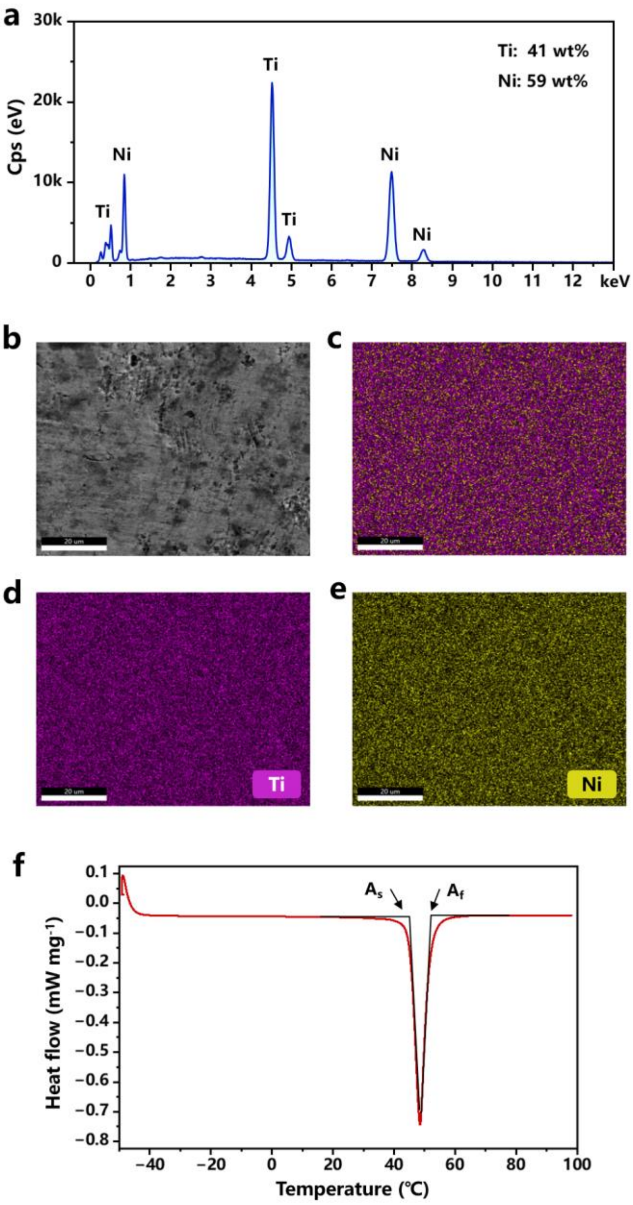

Figure S25. (a) EDS spectrum of the shape memory NiTi alloy (T_g ~ 50°C). (b) Surface morphology of the shape memory NiTi alloy. (c-e) Element distribution of Ti and Ni. (f) DSC curves of the shape memory NiTi alloy (T_g ~ 50°C). EDS analysis reveals that Ni accounts for 41.5% and Ti accounts for 58.5%, and the element distribution is uniform. DSC curve reveals that the martensitic-austenite transformation happens above the austenite start temperature (A_s, 45°C), and all deformation completes below the austenite finish temperature (A_f, 52°C).

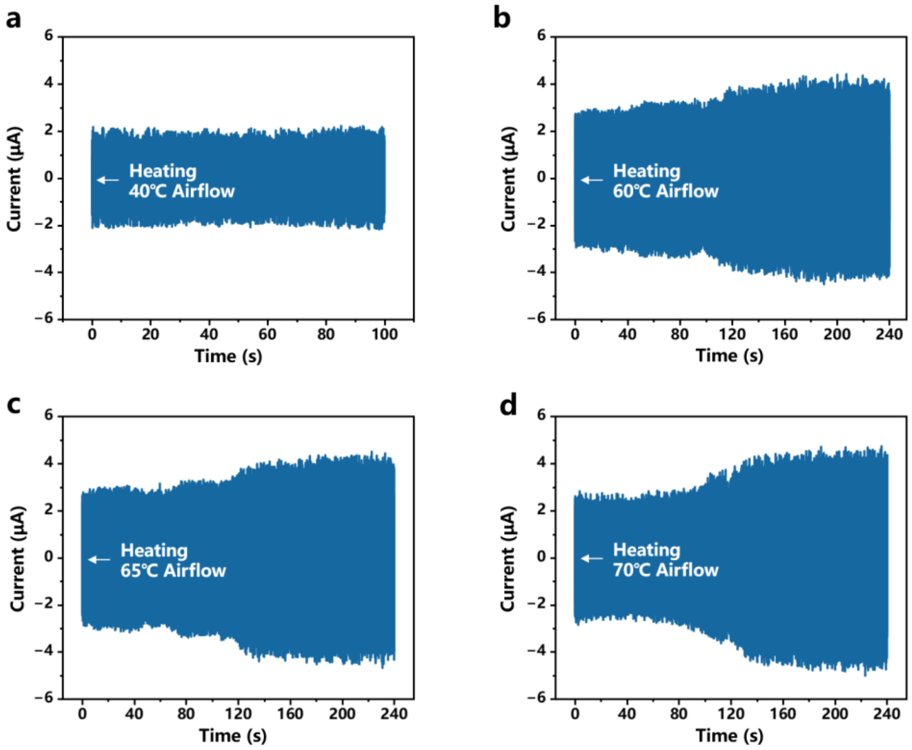

Figure S26. Current response waveform of RW-TENG toward various wind temperature stimulations (T_g of SMA: ~ 50°C).

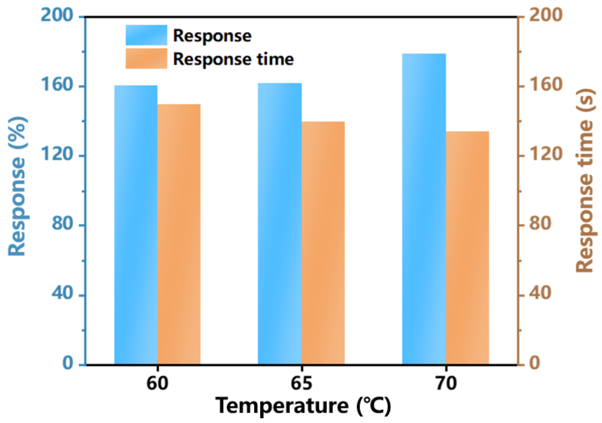

Figure S27. Comparison of the current response and response time of RW-TENG (T_g of SMA: ~ 50°C).

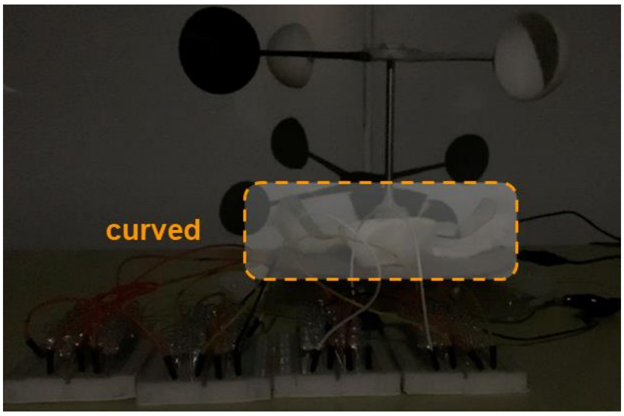

Figure S28. RW-TENG-based self-powered wind temperature alarm system under "curved" state.

Supporting Note S1: The charge transfer process and potential distribution of RW-TENG during operation.

A physical model based on Maxwell equations has been created to validate the charge transfer process of RW-TENG theoretically in one typical cycle, which is similar to conventional wind-driven TENGs with freestanding working mode. Figure N1 displays the sectional view of the device, and the two electrodes (A/B) are connected in the open-circuit state. It is assumed that any overlap between the rotator and the electrode can be modeled as a parallel plate capacitor without considering its edge effect, as the thickness of FEP is considerably less than its width [1].

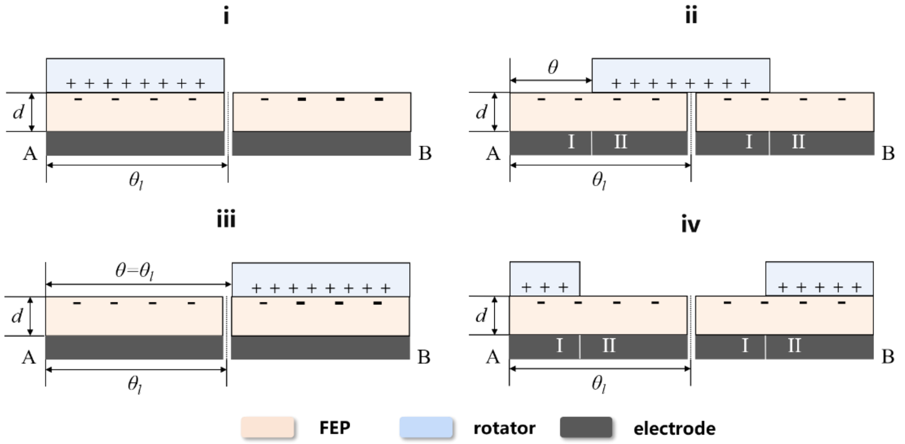

Figure N1. The charge transfer process of RW-TENG during operation.

Suppose that the surface charge density of FEP (ρ_{FEP}) is -σ, and the tribo-charge density on the rotator's surface (ρ_{Rotator}) changes with the electrostatic balance process when the rotator slides. In addition, suppose that the thickness of FEP is d, the sliding angle of the rotator is θ, and the center angle of electrode A (or B) is θ_l. The influence of the stator's air gap (other than the insulation performance) is ignored.

According to the model assumption, under state (i), the electric field generated by the tribo-charges in the dielectric is a uniform electric field. The upper surface of electrodes A and B induce negative and positive charges, respectively. Using the Gauss theorem, the open-circuit voltage can be calculated from Equation (S1), by setting the infinite point as zero potential.

$$V_{\mathrm{OC}}(\theta=0)=U_{\mathrm{A}}(\theta=0)-U_{\mathrm{B}}(\theta=0)=\frac{d\sigma}{\varepsilon_0\varepsilon_r}-(-\frac{d\sigma}{\varepsilon_0\varepsilon_r})=\frac{2d\sigma}{\varepsilon_0\varepsilon_r} \quad \text{(S1)}$$

where ε_0 is the vacuum dielectric constant (ε_0=8.85×10^{-12} F/m), and ε_r is the relative dielectric constant of FEP. Similarly, under state (iii), the open-circuit voltage can be calculated from Equation (S2).

$$V_{\mathrm{OC}}(\theta=\theta_l)=-V_{\mathrm{OC}}(\theta=0)=-\frac{2d\sigma}{\varepsilon_0\varepsilon_r} \quad \text{(S2)}$$

In addition to the two unique states (i) and (iii), the general circumstances during the operation

are considered as follows. Using state (ii) as an example, the potential difference distribution is analyzed during the sliding of RW-TENG from state I to state (iii) (i.e., $0 < \theta < \theta_l$). At this time, electrodes A and B are divided into two areas: I and II. The rotator covers area II of electrode A and area I of electrode B. The rest area (not covered) is only affected by the negative tribo-charges on the FEP surface. As a result, area I of electrode A induces positive charges, and the charge density can be calculated from Equation (S3).

$$\rho_{\mathrm{A(I)}} = \sigma \tag{S3}$$

Considering that the total charge in electrode A is 0, the remaining negative charges are all distributed in area II, and the charge density is shown in Equation (S4).

$$\rho_{\mathrm{A(II)}} = -\sigma \cdot \frac{\theta}{\theta_l - \theta} \tag{S4}$$

Similarly, area I and II in electrode B have the following surface charge density (Equation (S5)).

$$\rho_{\mathrm{B(I)}} = -\sigma \cdot \frac{\theta_l - \theta}{\theta}, \qquad \rho_{\mathrm{B(II)}} = \sigma \tag{S5}$$

At this time, the surface positive charges on the rotator are redistributed, depending on how electrode A and B surface charges have changed. According to the assumption of parallel plate capacitor, the rotator's surface is approximately equipotential, so the potential difference between the rotator's surface and the electrodes can be calculated from Equation (S6).

$$U_{\mathrm{Rotator}} - U_{\mathrm{A}} = \frac{d}{\varepsilon_0 \varepsilon_r} \cdot \frac{\theta}{\theta_l - \theta} \cdot \sigma, \qquad U_{\mathrm{Rotator}} - U_{\mathrm{B}} = \frac{d}{\varepsilon_0 \varepsilon_r} \cdot \frac{\theta_l - \theta}{\theta} \cdot \sigma \tag{S6}$$

Therefore, the open-circuit voltage between A and B is shown in Equation (S7).

$$V_{\mathrm{OC}}(\theta) = U_{\mathrm{A}}(\theta) - U_{\mathrm{B}}(\theta) = \frac{d\sigma}{\varepsilon_0 \varepsilon_r} \left(\frac{\theta_l - \theta}{\theta} - \frac{\theta}{\theta_l - \theta} \right) \tag{S7}$$

When area I equals area II, the open-circuit voltage can be calculated from Equation (S8).

$$V_{\mathrm{OC}}\left(\frac{\theta_l}{2}\right) = U_{\mathrm{A}}\left(\frac{\theta_l}{2}\right) - U_{\mathrm{B}}\left(\frac{\theta_l}{2}\right) = \frac{d\sigma}{\varepsilon_0 \varepsilon_r} \left(\frac{\theta_l - \frac{\theta_l}{2}}{\frac{\theta_l}{2}} - \frac{\frac{\theta_l}{2}}{\theta_l - \frac{\theta_l}{2}} \right) = 0 \tag{S8}$$

Based on the same method, during the transition from state (iii) to state (i) (i.e., $\theta_l < \theta < 2\theta_l$), the open-circuit voltage between A and B is shown in Equation (S9).

$$V_{\mathrm{OC}}(\theta) = U_{\mathrm{A}}(\theta) - U_{\mathrm{B}}(\theta) = \frac{d\sigma}{\varepsilon_0 \varepsilon_r} \left(\frac{\theta - \theta_l}{2\theta_l - \theta} - \frac{2\theta_l - \theta}{\theta - \theta_l} \right) \tag{S9}$$

Supporting Note S2: Calculation method of the output performance when RW-TENG is connected with external load.

RW-TENG is a capacitive device with high input impedance. If the physical model in Note S1 is adopted, the internal intrinsic capacitance C_T can be viewed as the series of $C_{Rotator-A}$ (the capacitance between electrode A and the rotator) and $C_{Rotator-B}$ (the capacitance between electrode B and the rotator), as shown in Equation (S10).

$$C_T = \frac{1}{\frac{1}{C_{Rotaror-A}} + \frac{1}{C_{Rotaror-B}}} = \frac{\varepsilon_0 \varepsilon_r}{d} \cdot \frac{\tilde{A}(\theta_l - \theta) \cdot \tilde{A}(\theta)}{\tilde{A}(\theta_l - \theta) + \tilde{A}(\theta)} \quad \text{(S10)}$$

where $\tilde{A}(\theta)$ and $\tilde{A}(\theta_l - \theta)$ represent the area of overlapping parts of the rotator and electrode A and B, respectively. The output of RW-TENG is approximately sinusoidal (Figure 1e-g). Therefore, when the external circuit is connected in series with the resistance load Z_R=R, the load current (I_R) of RW-TENG can be calculated from Equation (S11).

$$I_R = \frac{Z_T}{Z_T + Z_R} \cdot I_{SC} = \frac{1}{\sqrt{1 + (2\pi f \cdot R \cdot C_T)^2}} \cdot I_{SC} \quad \text{(S11)}$$

As a result, Equation (S12) represents the calculation expression for the average power density.

$$P_{Density} = \frac{I_{SC(m)}^2 \cdot R}{2A} \quad \text{(S12)}$$

where $I_{sc(m)}$ is the short-circuit current's peak value, R is the external circuit resistance, and A is the total area of the stator.

In addition, when the device can obtain the maximum power density, the external circuit load matches the internal resistance, as shown in Equation (S13).

$$R_m = \frac{1}{2\pi f \cdot C_T} \quad \text{(S13)}$$

The equation above shows that the impedance of RW-TENG is closely related to parameters including rotational frequency f and dielectric thickness d. When the wind speed decreases, the frequency f decreases, and the match resistance R_m increases. Furthermore, as shown in Figure N2, we further hypothesize that an air gap (average thickness of d_0) exists between the negatively charged FEP surface and the positively charged rabbit fur surface, due to the loose structure on the surface of rabbit fur. As for soft-contact working mode, the dielectric thickness should be therefore expressed as:

$$d = d_1 + d_0 \cdot \varepsilon_r \quad \text{(S14)}$$

while the dielectric thickness should be expressed as for direct-contact working mode:

$$d = d_1 \tag{S15}$$

Therefore, when soft-contact mode is employed, the dielectric thickness d increases, $C_{\text{Rotator-A/B}}$ decreases, and the match resistance R_m increases.

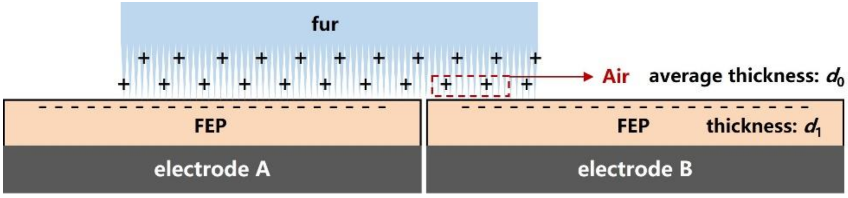

Figure N2. Schematic diagram of the intrinsic capacitance of RW-TENG.

Supporting Note S3: The working principle of the oil-immersed transformer cooling system.

As shown in Figure N3, the cooling system of the oil-immersed transformer consists of fans, oil pumps, oil-flow relay, and heat sink. The heat from winding and iron core is cooled by transformer oil that flows to the cooler body through the top oil pipe. Heat in transformer oil is dissipated to the outside through the cooling fans. The cooled low-temperature transformer oil returns to the oil tank through the oil pump and the oil-flow relay to cool the windings and iron core again. Among them, the oil-flow relay can display changes in oil flow in the cooling system to monitor the operation of the oil pump.

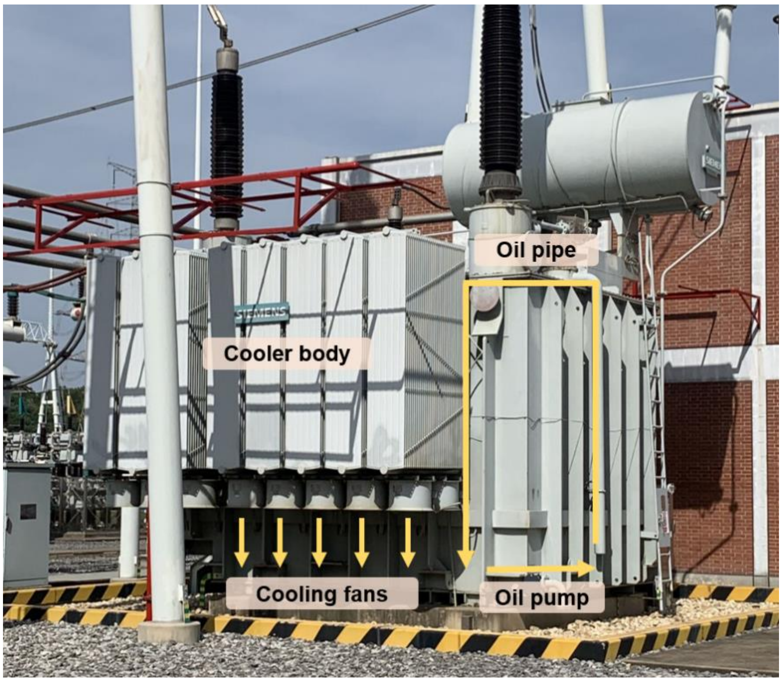

Figure N3. Schematic diagram of the cooling system operation principle. The photograph was taken at the 500 kV main transformer.

Supporting Note S4: The comparison of detection limit between our proposed self-powered wind sensor and commercial wind sensor

In this work, the industrial digital anemometer (TA8161) was employed to measure the wind speed of the blower output, which can be utilized as a wind-temperature sensor as well. Take such commercial wind sensor as an example, the measurement error of wind speed is ± 3%, and the detection limit of wind speed is 0.1 m s^{-1}. Additionally, the temperature measurement error is ± 2%, and the detection limit of wind temperature is 0.1℃. Herein, our proposed RW-TENG with SMA of the "curved" state demonstrated the sensitivity of 0.15 μA m^{-1} s and 0.526 μA m^{-1} s of the "flattened" state as a self-powered wind-speed sensor, while the device demonstrated the sensitivity of 2.22℃ s^{-1} as a self-powered airflow temperature sensor.

Further, we estimated the detection limit of our proposed wind sensor by the following formula[2]:

$$\mathrm{DL} = 3 \times \frac{\sqrt{\frac{\Sigma(y_i - y)^2}{n}}}{k} \tag{S16}$$

where y_i is the recorded sensor response, y is the average value of y_i, k is the slope of fitted curve, and DL is the detection limit. In addition, considering the fluctuation of measured output current, the response data with maximum measurement error at various wind speed and temperature points was selected as y_i. Our wind speed sensor demonstrated a detection limit of **2.15 m s^{-1}**, and our wind temperature sensor demonstrated a detection limit of **15.56℃**, which is lower than the accuracy of battery-powered commercial sensors. Even so, determined by the working mechanism of RW-TENG, this detection limit is competent for most applications, and our device exhibits benefits including self-power, round-the-clock operation, and minimal maintenance expenditures over commercial sensors. Meanwhile, our proposed device can precisely identify the alarm wind temperature thanks to SMA's tunable phase transition temperature, which has wide application prospects in industrial scenarios.

References:

[1] G. Zhu, J. Chen, T. Zhang, Q. Jing, Z. L. Wang, *Nat Commun* 2014, *5*, 3426.

[2] P. Wu, Y. Li, S. Xiao, D. Chen, J. Chen, J. Tang, X. Zhang, *ACS Appl. Mater. Interfaces* 2022, *14*, 48200.
